# Does power ultrasound affect hydrocarbon Ionomers?

**DOI:** 10.1016/j.ultsonch.2021.105588

**Published:** 2021-05-07

**Authors:** Michael Adamski, Nicolas Peressin, Emmanuel Balogun, Bruno G. Pollet, Steven Holdcroft

**Affiliations:** a Holdcroft Research Group, Department of Chemistry, Simon Fraser University, 8888 University Drive, Burnaby, BC V5A 1S6, Canada; b Hydrogen Energy and Sonochemistry Research Group, Department of Energy and Process Engineering, Norwegian University of Science and Technology (NTNU), NO-7491 Trondheim, Norway

**Keywords:** Fuel cell, Electrolyzer, Hydrocarbon ionomer, Catalyst layer, Power ultrasound, Sonochemistry, SPPB, HMT-PMBI, Catalyst inks

## Abstract

The effect of low-frequency high-power ultrasound on hydrocarbon-based ionomers, cation exchange sulfonated phenylated polyphenylene (sPPB-H^+^) and anion exchange hexamethyl-*p*-terphenyl poly(benzimidazolium) (HMT-PMBI), was studied. Ionomer solutions were subjected to ultrasonication at fixed ultrasonic frequencies (*f* = 26 and 42 kHz) and acoustic power (*P*_acous_ = 2.1 – 10.6 W) in a laboratory-grade ultrasonication bath, and a probe ultrasonicator; both commonly employed in catalyst ink preparation in research laboratory scale. Power ultrasound reduced the polymer solution viscosity of both hydrocarbon-based ionomers. The molecular weight of sPPB-H^+^ decreased with irradiation time. Changes in viscosity and molecular weight were exacerbated when ultrasonicated in an ice bath; but reduced when the solutions contained carbon black, as typically used in Pt/C-based catalyst inks. Spectroscopic analyses revealed no measurable changes in polymer structure upon ultrasonication, except for very high doses, where evidence for free-radical induced degradation was observed. Ionomers subjected to ultrasound were used to prepare catalyst layers and membrane electrode assemblies (MEA)s. Despite the changes in the ionomer described above, no significant differences in electrochemical performance were found between MEAs prepared with ionomers pre-subjected to ultrasound and those that were not, suggesting that fuel cell performance is tolerant to ionomers subjected to ultrasound.

## Highlights

• The effects of an ultrasonicating bath and probe on ionomer solutions was examined.
• Two hydrocarbon polymers, sPPB-H^+^ and HMT-PMBI, were ultrasonicated for 0–480 min.
• Power ultrasound caused a stepwise reduction in viscosity and molecular weight.
• Chain scission likely occurs due to the mechanical forces of solution cavitation.
• Fuel cell electrochemical characteristics were unchanged by ionomer ultrasonication.

## Graphical abstract

**Figure:**
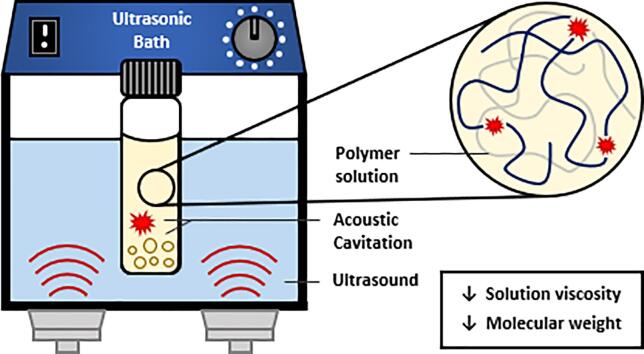

## 1 Introduction

**Fig. 1:**
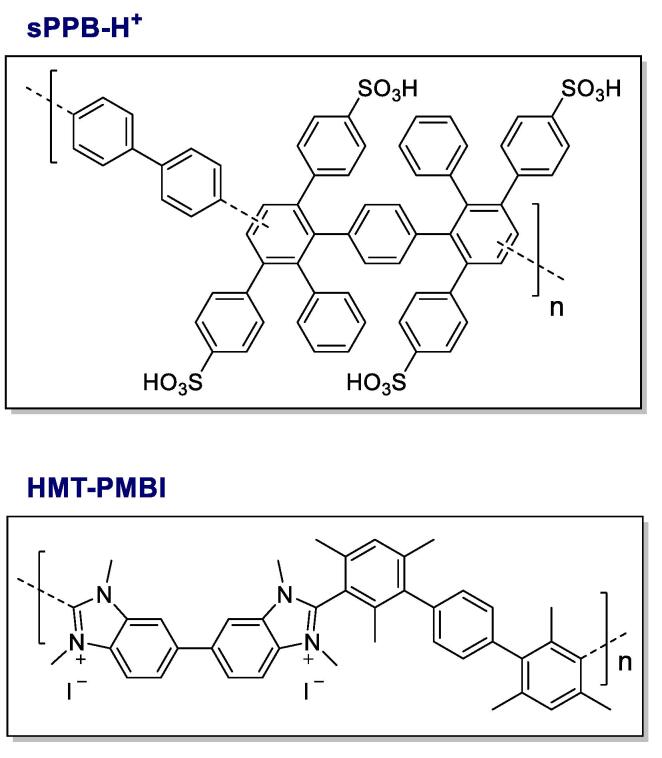
Chemical structures of sPPB-H^+^, and HMT-PMBI. The latter anion exchange polymer may contain various anionic counterions when fully functionalized (100% degree of methylation), such as iodide (as pictured).

Low temperature fuel cells and electrolyzers are leading power conversion devices for mobile and stationary power systems. Both use a solid polymeric membrane as the central ion exchange medium and contain additional ion-containing polymer (ionomer) dispersed throughout the catalyst layer (CL) as a binding agent and transporter of ions and water. In order to meet cost targets set by the US Department of Energy [1], and the EU Fuel Cells and Hydrogen Joint Undertaking [2], potential substitutes to incumbent perfluorosulfonic acid (PFSA)-based proton exchange membranes (PEMs) and ionomers (e.g., Nafion®) have been extensively researched and developed over the last decade [3], [4], [5], [6]. It has become evident that both cation and anion conducting hydrocarbon-based polymeric materials represent viable alternatives for polymer electrolyte membrane fuel cells (PEMFCs) and water electrolyzers (PEMWEs) - due to their low cost, ease of synthesis, reduced environmental concerns, and lower rates of gas permeability crossover [3], [6], [7], [8], [9]. For example, cation-conducting sulfonated phenylated poly(phenylene)s such as sPPB-H^+^ (Fig. 1) have shown promise in fuel cell applications as both membranes and ionomers in the catalyst layer [10], [11], and are currently being investigated in water electrolyzers. Similarly, hexamethyl-*p*-terphenyl poly(benzimidazolium) HMT-PMBI (Fig. 1) has been employed as a hydrocarbon-based anion exchange polymer, applicable to anion exchange membrane fuel cells (AEMFC) and water electrolyzers (AEMWE) [8], [12].

Unfortunately, there is limited information in the literature on critical parameters for the use of hydrocarbon ionomers, compared to the large number of reports on PFSAs. For instance, while the optimal catalyst ink composition for PEMFCs and PEMWEs using Nafion® is well-established, there are very few reports which have evaluated catalyst ink composition using hydrocarbon PEMs [11], [13]. The understanding of the role of anion exchange membranes (AEMs) with respect to AEMFCs and AEMWEs is even less clear, because there has been no long-standing commercial reference material *akin* to that of Nafion® [8], [14]. In order to continue progressing the utility of hydrocarbon-based materials, additional fundamental research on both membranes and ionomer solutions (e.g., catalyst inks) is required.

Researchers in a typical academic research laboratory investigating the fabrication of MEAs for fuel cell and water electrolyzer applications often employ ultrasound during catalyst ink preparation (dispersion), yet, the effect of ultrasound on components of the ink, particularly the ionomer, is largely unknown [15], [16], [17]. This is surprising given the effects of power ultrasound (20 kHz – 2 MHz) in polymerization and depolymerization has been the subject of study for many years [18], [19], [20], [21], [22], [23]. It is known that ultrasonic irradiation accelerates polymerization, as well as depolymerization, by thermal activation and free radical formation, and that it can facilitate the synthesis of both high and low molecular weight polymers. Within the sonochemistry community (the use of power ultrasound in chemistry), it is accepted that power ultrasound leads to polymer degradation and decomposition, and to reductions in molecular weight (MW) and solution viscosity (*η*) [20], [24], [25], [26].

In the field of sonochemistry, acoustic cavitation is known to cause emulsification, molecular degradation, luminescence and erosion, principally associated with the collapse of high energy cavitation bubbles [27]. It is estimated that extraordinarily high localized temperatures (up to 10,000 K) and pressures (up to 5,000 atm; 500 MPa) are generated, together with a collision density of 1.5 kg·cm^−2^ and pressure gradients of up to 2 TPa·cm^−1^, with lifetimes < 0.1 μs, causing localized heating rates above 10^9–10^ K·s^−1^. These conditions are formed during collapse of a cavitation bubble [28], [29], [30], [31]. In addition, it is documented that highly reactive radical species are formed via water sonolysis induced by acoustic cavitation [22], [23], [27]. Sonolysis involves the homolytic cleavage of water to generate OH•, HO_2_•, H• and O•, any of which may react with and degrade compounds, or in the case of polymers, attack C–C bonds in the polymer backbone [22], [23], [32], [33]. The practical result of cavitation is three-fold: (i) generation of high local temperatures, (ii) physical forces, and (iii) reactive chemical species [30], [33]. The intensity of the cavitation effect varies with the (liquid) solvent medium employed, as well as its temperature [28].

The morphology and electrochemistry of catalyst layers is complex [34], [35]. Minor changes to their components, such as molecular weight of the ionomer [36], or concentration [11], [13], may have a profound effect on overall device performance. In order to shed light on the effect of ultrasonication on the catalyst inks, and the potential impact on the electrochemical devices prepared, it is informative to probe the effect of ultrasonication on the ionomers. Recently Adamski *et. al.* [17] investigated the effect of power ultrasound on Nafion® dispersions, from which catalyst inks are typically prepared. The viscosity of Nafion® dispersions decreased with increasing ultrasonication time, potentially due to gradual, stepwise depolymerisation (degradation) of the ionomer [17]. Similar observations were previously described by Momand [37], of the Pollet research group (University of Birmingham, UK) in 2013.

In this investigation, the effects of power ultrasound (*f* = 26 and 42 kHz, *P*_acous_ = 2.1 – 10.6 W, ultrasonic bath and probe) on two very different hydrocarbon-based ionomers (a PEM and AEM), sPPB-H^+^ and HMT-PMBI, were examined. Various concentrations of polymer solution were evaluated under differing irradiation durations (*t_US_*) both at ambient (room temperature), and cooled (ice bath) conditions. Investigations of the effect of ultrasound were complemented with the studies of the effect of probe ultrasonication, and rapid stirring (1,000 RPM) in the absence of ultrasound (*silent* conditions), because these represent alternative methods for catalyst ink preparation. In addition, the effect of added carbon black was probed because such mixtures are representative of typical catalyst inks prepared for fuel cells and water electrolyzers [11], [12], [13]. The work is complemented by fabricating and characterizing catalyst inks containing sPPB-H^+^ that were ultrasonicated prior to use in MEAs and fuel cells.

## 2 Experimental methods

### 2.1 Polymers

Both the cation exchange polymer sPPB-H^+^ (IEC = 3.23 ± 0.04 meq·g^−1^) [10], and anion exchange polymer HMT-PMBI (degree of methylation = 92.7 ± 0.4) [8], were synthesized according to previously published methodologies [8], [10]. From pure polymer resins, stock solutions of sPPB-H^+^ and HMT-PMBI (6.67% w/w in MeOH) were prepared, which were diluted with appropriate amounts of MeOH and deionized H_2_O (18.2 mΩ) to give 1.00, 0.30, and 0.15 wt% solutions of each polymer in 3:1 v/v MeOH/H_2_O. Each solution was then partitioned into 27.0 ± 0.1 mL samples contained within 30 mL, 9 cm tall VWR® glass vials (short form style with phenolic cap on). The samples, labelled according to the constituent polymer and concentration (e.g., sPPB-H^+^ 0.15 wt%), were subject to individual experiments as described below. In addition, to several 0.30 wt% polymer solutions was added 0.70 wt% HSA (high surface area) carbon black (Ketjenblack EC-300 J, FuelCellStore), under rapid stirring (1,000 RPM for 30 min). The resulting samples were comprised of 0.30 wt% dissolved ion exchange polymer (ionomer), 0.70 wt% inorganic solids (carbon black), and 99.0 wt% solvent, effectively emulating a common hydrocarbon PEM catalyst ink [13].

### 2.2 Ultrasound and rapid stirring

Ultrasonication in a water bath was performed using a 42 kHz Bransonic B1510R-MT Ultrasonic Cleaner filled to its marked operating level (1,300 mL) with either water at room temperature (19.0 ± 0.1 °C), or with an ice bath comprised of water and 500 mL crushed ice (equilibrated temperature = 0.3 ± 0.1 °C). The acoustic power was measured calorimetrically to be 2.1 ± 0.1 W. Aluminum foil tests were performed using a 15 × 80 mm strip of aluminum foil immersed in the center of 27.0 mL of the indicated solution. Aluminum foil samples, and polymer solutions were suspended 7 cm deep in the center of the ultrasonication bath such that the sample meniscus was level with the ultrasonication bath water height (operating level). Ultrasonication was performed for 0 – 480 min durations, as indicated.

Ultrasonication of the ionomer samples using a sonicating probe was performed using a Hielscher UP200St Ultrasonic Homogenizer equipped with a titanium sonotrode (7 mm bottom diameter, Hielscher S26d7D) set to 10 W and 25.91 kHz at 100% pulse (operating continuously). The acoustic power was measured calorimetrically to be 10.6 ± 0.2 W. The sonotrode was inserted directly into the sample to a depth of 80 mm. Each 27.0 ± 0.1 mL sample, contained within 30 mL, 9 cm tall VWR® glass vials (short form style with phenolic cap on), was fixed in the center of a small ice bath (3:1 crushed ice/water in a 250 mL beaker), and ultrasonicated following a 5 min temperature equilibration (0.3 ± 0.1 °C). Ultrasonication was performed for 0 – 20 min durations.

Rapid stirring (1,000 RPM) was performed using a Teflon-coated stirring bar and IKA RCT Basic Magnetic Stirring Plate set to 1,000 RPM. After ultrasound or rapid stirring, polymer solutions were equilibrated to room temperature with continuous stirring (200 RPM) until rheology measurements were performed. Prior to characterizations, solutions containing carbon black were filtered through 0.45 μm PES sterile syringe filters (VMR). A summary of experiments performed is given in Table 1.

**Table 1:** Ultrasound, stirring, and high shear mixing experiments performed using sPPB-H^+^ and HMT-PMBI solutions of varying concentrations (1.00, 0.30, and 0.15 wt% in 3:1 MeOH/H_2_O v/v).

Treatment	Duration (min)	Initial Temperature (°C)	Stirring (rpm)
None (control)	N/A	19.0 ± 1.0	200
Bath ultrasonication	5	19.0 ± 0.1^a^	200, during initial sample prep
	10		
	20		
	40		
	60		
	90		
	120		
	240		
	480		
Bath ultrasonication in an ice bath	5	0.3 ± 0.1	200, during initial sample prep
	10		
	20		
Bath ultrasonication with added carbon black in the polymer solution^b^	0	19.0 ± 0.1^a^	5 min @ 1,000, during addition of carbon black
	5		
	10		
	20		
Bath ultrasonication with added carbon^b^In an ice bath	0	0.3 ± 0.1^c^	5 min @ 1,000, during addition of carbon
	5		
	10		
	20		
Probe ultrasonication	0	0.3 ± 0.1	200, during initial sample prep
	5		
	10		
	20		
Rapid stirring	1,440 (24 h)	19.0 ± 1.0	1,000

a Ultrasonication caused heating of the bath (and sample) over time, up to ~ 47 °C after 8 h.

b Carbon black was filtered out of solution following sonication, prior to solution characterization.

c Ultrasonication caused melting of the ice bath over time (approx. 10 min) during which bath temperature remained low (<1.0 °C), and after which gradual heating (up to 1.8 °C) was observed after 20 min.

### 2.3 Rheology, molecular weight determination, and NMR spectroscopy

The effective shear viscosity of polymer solutions was measured on an Anton Paar MCR 102 Rheometer equipped with a standard cup and concentric cylinder bob geometry. A cap was employed to limit solution evaporation. Each polymer solution sample was equilibrated to 20.0 ± 0.1 °C (10 min) and held at that temperature for the duration of the rheology measurements. A low viscosity mode measurement profile was utilized. The shear rate was ramped linearly from d(γ) · dt^−1^ = 1 to 100 Hz with point durations ramped linearly from 10 to 1 s. All assessments were performed thrice in series, separated by a 1-minute measurement pause. Samples were hence each measured in triplicate. All errors provided are measurement standard deviations. The instrumental error was 0.1 mPa⋅s.

Both sPPB-H^+^ and HMT-PMBI polymer solutions exhibited Newtonian behavior. Minor increases in polymer solution viscosity were observed during sample characterization, which may have been due to gradual solvent evaporation. To determine the viscosity of a given polymer solution, the zero-shear viscosity was approximated by fitting viscosity data between 20 and 100 Hz of a given rheology sweep. Relative solution viscosities (*η*_relative_) were calculated using Eq. (1), where *η*_solution_ and *η*_solvent_ are the measured average shear viscosities of the polymer dispersion and pure solvent, respectively. From this inherent viscosity (*η*_inherent_) was determined using Eq. (2), where *c* is the concentration of assessed polymers in solution in g·dL^−1^: 1.00 wt% *c* = 1.00 g·dL^−1^; 0.30 wt% *c* = 0.30 g·dL^−1^; 0.15 wt% *c* = 0.15 g·dL^−1^.

(1) $$\eta_{\mathrm{relative}}=\frac{\eta_{\mathrm{solution}}}{\eta_{\mathrm{solvent}}}$$

(2) $$\eta_{\mathrm{inherent}}=\frac{{ln(\eta }_{\mathrm{relative}})}{c}$$

Molecular weight parameters of polymers were determined by size exclusion chromatography (SEC) using a Malvern Omnisec Resolve GPC system equipped with a Viscotek D6000M primary column and Viscotek D3000 secondary column using HPLC grade DMF (containing 0.01 M LiBr) as eluent. Narrow molecular weight distribution polystyrene standards (PS; *M*_w_ = 105,982, *M*_n_ = 101,335 g·mol^−1^) were used to calibrate the system. Calibration was verified by measuring a standard with a wider dispersity (PS; *M*_w_ = 247,581, M_n_ = 104,485 g·mol^−1^).

^1^H NMR spectra of samples normalized to 20 mg·mL^−1^ (polymer in solvent) were obtained on either a Bruker AVANCE III 400 MHz or Bruker AVANCE III 500 MHz, both running IconNMR under TopSpin 2.1, as indicated. Additional information regarding SEC and ^1^H NMR characterization and parameters is provided in the Supplementary Information (SI).

### 2.4 Determination of ultrasonic power

The ultrasonic (or acoustic) power of the ultrasonication bath and probe were determined calorimetrically using the methods of Margulis *et. al.* [38] and Contamine *et. al*. [39], and using Eq. (3), where (d*T*/d*t*)*_t_*_=0_ is the gradient of the water temperature per unit of ultrasonication time (at *t* = 0) in K·s^−1^, *m* is the mass of the water used in grams, and *C_p_* is the specific heat capacity of water as 4.186 J·g^−1^·K^−1^.

(3) *P*_acous_ = (d*T*/d*t*)*_t_*_=0_ × *m* × *C*_p_

The calorimetric method consists of measuring the heat dissipated in a volume of water, taking into account the water heat capacity (*C*_p_) in which the acoustic energy is absorbed. This method assumes that all absorbed acoustic energy is transformed into heat. From the calorimetric experiments, the acoustic power, *P*_acous_ in W was determined. Under conditions employed in this work, the *P*_acous_ was 2.1 ± 0.1 W for the ultrasonication bath, and 10.6 ± 0.2 W for the ultrasonication probe.

### 2.5 Dosimetry

Measuring the formation of radicals in an aqueous solution during ultrasonication is challenging due to the short lifespan of a radical. We used the Weissler dosimetry method to determine the rate of formation of hydroxyl radicals (OH•) during ultrasonication [30]. Aqueous 0.1 mol·L^−1^ potassium iodide (KI) solutions were ultrasonicated for 5, 10, and 20 min, as described by Iida *et. al.* [40]*,* Son *et. al.* [41], and La Rochebrochard d’Auzay *et. al.*, [42] to determine OH• radical concentrations. The formation of I_3_^−^ was monitored by UV–Vis spectrophotometry at a wavelength (*λ*) of 355 nm using a molar absorptivity (ε) of 26,303 dm^3^·mol^−1^·cm^−1^ [42]. In this process, iodide ions I^−^ are oxidized by OH• to yield iodine atoms (I, as per Eq. (4)). Iodine atoms react with I^−^ to produce I_2_^−^ (Eq. (5)) which subsequently yields I_2_ (Eq. (6)). Molecular iodine reacts with excess I^−^ to form triiodide ions, I_3_^−^ (Eq. (7)). The absorbance of I_3_^−^ may then be measured using a UV–Vis spectrometer. This method also allows the determination of the rate of triiodide anion formation *ν*(I_3_^−^) (mol·s^−1^), and thus the rate of OH• formation. That is, assuming that *ν*(I_3_^−^) = *ν*(OH•).

(4) OH• + I^−^ →OH^–^ + I

(5) I + I^−^ →I_2_^−^

(6) 2I_2_^−^→ I_2_ + 2I^−^

(7) I_2_ + I^−^ →I_3_^−^

A detailed description of the experimental procedure is provided in the SI. To represent the energy-specific yield for this dosimetry, the sonochemical efficiency (SE) value can be determined in μmol·kJ^−1^ per Eq. (8) [43]. SE represents the ratio of the number of the reacted molecules towards the ultrasonic energy (determined calorimetrically). The SE was calculated via Eq. (8), where [I_3_^−^] (μmol·L^−1^) is the triiodide concentration, *V* (L) is the solution volume, *P*_acous_ (kW) is the acoustic power, and *t* (s) is the ultrasonication time.

(8) *SE* = ([I_3_^−^] × *V*) / (*P*_acous_ × *t*)

### 2.6 Membrane-electrode-assembly and fuel cell operation

Six different batches of catalyst inks were prepared, consisting of a homogeneous mixture of Pt/C catalyst powder (Tanaka, TEC10E50E, lot 109–0111, 46.4% Pt), de-ionized water, methanol (reagent grade, Fischer Scientific), and polymer (ionomer) solution. Three PFSA and three hydrocarbon inks were prepared, containing Nafion® D520 (Ion Power Inc.) PFSA ionomer, and sPPB-H^+^, respectively. First, 1.00 wt% polymer solutions were pre-sonicated by using either an ultrasonication bath (US-Bath), or an ultrasonication probe (US-Probe), for 20 min. A third, reference solution was used without any ultrasonication. Procedures for catalyst ink formulation and preparation of membrane electrode assemblies (MEAs) for fuel cell characterization are detailed in the SI. All MEAs contained a catalyst loading of 0.4 mg Pt·cm^−2^ in both the anode and cathode. The electrochemical characterization techniques employed in this work have been previously reported by E. Balogun, *et. al*. [44] Electrochemical impedance spectroscopy was used to determine charge transfer resistance of the fuel cell under H_2_/O_2_ (anode/cathode) operation [45], and the ionic resistance of the catalyst layer under H_2_/N_2_ (anode/cathode) operation [46]. Three independently fabricated MEAs were characterized for each of the ultrasonic bath (US bath), ultrasonic probe (US probe), and reference catalyst ink samples (n = 3).

## 3 Results and discussion

Following irradiation with ultrasound or rapid stirring (1,000 RPM), each polymer solution was first equilibrated to room temperature. The ambient temperature of both the polymer solutions, and the ultrasonication bath water, was 19.0 ± 0.1 °C, and 0.3 ± 0.1 °C in an ice bath. The temperature of the ultrasonication bath was measured prior to and following each trial. In general, an increase in temperature with increasing exposure to ultrasound was observed, with bath temperatures reaching up to 45.8 ± 0.6 °C after 8 h. In contrast, experiments performed in an ice bath did not increase appreciably in temperature (remained at < 1.0 °C) until most of the ice had melted (approx. 10 min), eventually reaching 1.8 ± 0.1 °C after 20 min.

**Fig. 2:**
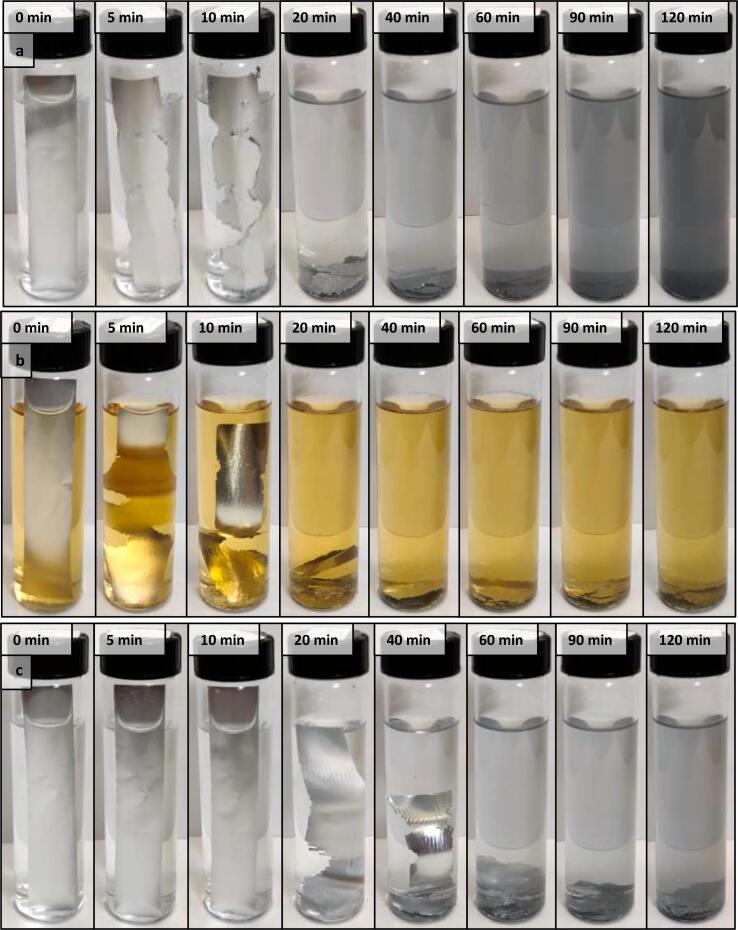
Damage caused to a 15x80 mm strip of aluminum foil immersed in a given solution and sonicated at a given temperature for 5–120 min. (a) in 3:1 MeOH/H_2_O beginning at ambient temperature (19.0 ± 0.1 °C); (b) in 3:1 MeOH/H_2_O containing 1.00 wt% sPPB-H^+^ beginning at ambient temperature (19.0 ± 0.1 °C); (c) in 3:1 MeOH/H_2_O in an ice bath (0.3 ± 0.1 °C).

An initial qualitative assessment of the cavitation phenomenon in 3:1 MeOH/H_2_O was performed using aluminum foil [33]. Into each of two sample vials was added 27.0 mL pure solvent and a 15x80 mm strip of aluminum foil. A third vial was prepared containing 27.0 mL of 1.00 wt% sPPB-H^+^ solution, instead of pure solvent, and a strip of foil. The resulting samples were ultrasonicated either at ambient temperature, or in an ice bath, as indicated, for 5–120 min durations, during which they were intermittently removed and photographed (Fig. 2). Pinholes were observed and cavitation was thus evident after just 5 min in samples ultrasonicated at ambient temperatures (Fig. 2a and b). In both cases, the aluminum foil showed signs of significant damage after 10 min, and had fragmented into smaller flakes after 20 min. This effect appeared to be slightly diminished when the solvent medium, pure 3:1 MeOH/H_2_O, was replaced with 1.00 wt% sPPB-H^+^ solution (Fig. 2b), from which it may be inferred that the presence of polymer dissolved in solution dampens, or absorbs, a portion of the applied ultrasonic energy.

When the aluminum foil experiment was performed in an ice bath (Fig. 2c), the rate of foil destruction was notably reduced (by approx. 20 min). This is likely because the temperatures at which cavitation reaches an intensity maximum in water and methanol are relatively high, 35 and 19 °C, respectively [28]. At ice bath temperatures (0.3 ± 0.1 °C), the relative cavitation intensity in either solvent medium is markedly lower: <70% of each respective maxima [28]. In addition, it is likely that the ice in the ice bath dampens the ultrasonic energy imparted onto the sample.

### 3.1 Rheology

**Fig. 3:**
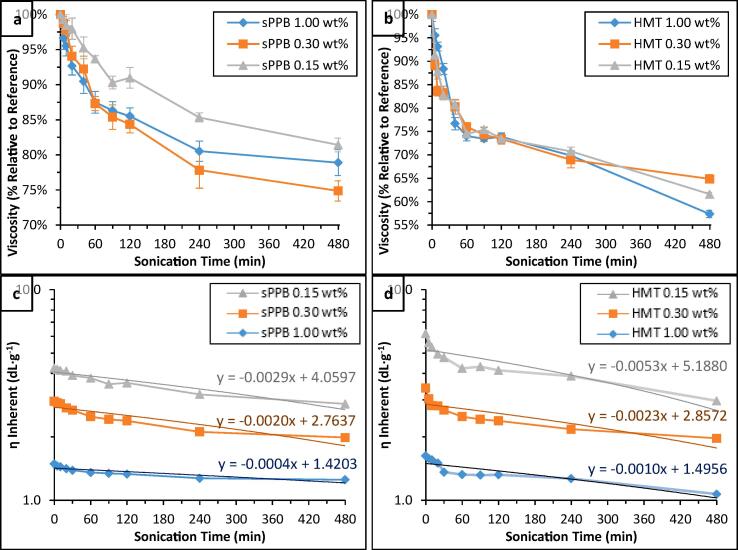
Normalized viscosities of (a) sPPB-H^+^ and (b) HMT-PMBI solutions (1.00, 0.30, and 0.15 wt% in 3:1 MeOH/H_2_O) following sample ultrasonication for 0 – 480 min at ambient temperature; calculated inherent viscosities and linear fits of (c) sPPB-H^+^ and (d) HMT-PMBI solutions. Data for HMT-PMBI 0.15 wt% (+1.0 dL·g^−1^) and 1.00 wt% (+0.1 dL·g^−1^) are offset vertically for visual clarity. Error bars represent the standard deviation of n = 3 unique samples.

The rheology of polymer solutions was individually measured at constant temperature (20 ± 0.1 °C). Within each series of sPPB-H^+^ and HMT-PMBI polymer solutions (1.00, 0.30, and 0.15 wt%), notable, stepwise reductions in viscosity were measured as a function of increasing sample ultrasonication time (see Fig. S1). When plotted as percentage values relative to each respective reference sample (*t_US_* = 0 min, viscosity = 100%; see Fig. 3a and b, and Fig. S2), it appeared as though sample concentration did not substantially affect the observed reductions in viscosity over time. In all cases, the rate of decline of the viscosity of the polymer solutions appeared to diminish with increasing ultrasonication time, particularly as *t_US_* > 60 min. Notably, the reductions observed in sPPB-H^+^ solution viscosity were less than that of HMT-PMBI solutions. For instance, after 120 min, solutions of HMT-PMBI showed up to 26.6 ± 1.1% reductions in viscosity, whereas for sPPB-H^+^ that value was 15.6 ± 1.3%. Similar observations were made when ultrasound experiments were extended to 480 min: HMT-PMBI showed up to 42.6 ± 0.8% reductions in viscosity, versus up to 25.1 ± 1.4% reductions in viscosity in the case of sPPB-H**^+^**. These data suggest that HMT-PMBI may be more susceptible to changes in solution, such as potential degradation, upon exposure to ultrasonication irradiation.

The experimental data were further probed by calculation of inherent viscosities, *η*_inherent_, of the polymer solution as per Eqs. (1), (2). Data are shown in Fig. 3c and d, with corresponding normalized values plotted in Fig. S3. In this case, a very similar trend was observed: increasing exposure to ultrasonication resulted in decreasing inherent viscosity of the polymer solution. The magnitude of reduction in inherent viscosity decreased with each step. The highest concentration sPPB-H^+^ and HMT-PMBI solutions (1.00 wt%) exhibited the smallest reductions in their overall inherent viscosity. This is evidenced by the calculated rates of reduction of *η*_inherent_ for both polymers, given by the respective slopes of the linear fits of the data (Fig. 3c and d). Low (0.15 wt%) and medium (0.30 wt%) concentration sPPB-H^+^ solutions exhibited rates of reduction of *η*_inherent_ which were ~ 7x (-0.0029 dL·g^−1^·min^−1^) and ~ 5x (-0.0020 dL·g^−1^·min^−1^) greater than that of the high concentration (1.00 wt%, −0.0004 dL·g^−1^·min^−1^) solutions. In the case of HMT-PMBI, these values were approximately 5x (0.15 wt%, −0.0053 dL·g^−1^·min^−1^) and 2x (0.30 wt%, −0.0023 dL·g^−1^·min^−1^) greater, respectively, than the 1.00 wt% solution (-0.0010 dL·g^−1^·min^−1^). Given that the high concentration (1.00 wt%) polymer solutions contain between 3x and ~ 7x more polymer by mass than the medium (0.30 wt%) and low (0.15 wt%) concentration samples, respectively, the dispersion of ultrasound energy across a larger number of macromolecules may equate to reduced degradation of each macromolecule, respectively. This in-turn would have the effect of slowing the rate of reduction of a solution’s inherent viscosity, if due to degradation, as observed in the case of the 1.00 wt% solutions.

In stark contrast to the ultrasonicated polymer solutions, samples treated with rapid stirring (1,000 rpm for 24 h) showed negligible changes in viscosity (Table 2, and Fig. S2 standalone circle markers). Similar results have previously been reported using Nafion® dispersions of differing concentrations (10, 5, and 2.5 wt%) [17]. Whereas ultrasonication in an ultrasonication bath for 60 min reduced the viscosity of Nafion® dispersions by up to 10.4 ± 1.8%, prolonged rapid stirring (1,000 RPM for 24 h) yielded negligible changes – a maximum decrease in viscosity of 2.3 ± 1.0% [17]. These data suggest that the use of rapid stirring, instead of ultrasound, is a viable means of dispersing the polymer solutions without inducing reductions in solution viscosity.

**Table 2:** Normalized viscosities of sPPB-H + and HMT-PMBI solutions following rapid stirring (1,000 rpm for 24 h at ambient temperature). Error is reported as the standard deviation of n = 3 unique samples.

Concentration	Viscosity (relative to reference)		
	1.00 wt%	0.30 wt%	0.15 wt%
sPPB-H^+^	97.4 ± 1.1%	99.4 ± 1.4%	103.9 ± 2.0%
HMT-PMBI	98.5 ± 0.5%	98.5 ± 1.1%	100.3 ± 0.8%

Fabrication of the catalyst inks for PEMFCs typically involves addition of appreciable amounts of Pt catalyst on carbon support, relative to the ionomer, followed by dispersion, or homogenization, via power ultrasound (e.g., in an ultrasonication bath). For instance, catalyst inks containing Nafion® D520 ionomer are typically prepared with 0.30 wt% ionomer and 0.70 wt% Pt/C [10], [13], whereas hydrocarbon-based catalyst inks range from 0.15 to 0.30 wt% ionomer, and 0.85 – 0.70 wt% Pt/C [11], [13], [47]. In both cases, 99.0 wt% of the ink is solvent/dispersant. While these values may differ in the case of AEMFC and AEMWE catalyst inks, the general procedures and excess of carbon black versus ionomer are maintained throughout [8], [12]. Ultrasound is typically applied for 10 – 120 min [11], [12], [48], [49], [50], and an ice bath may be employed in place of ambient water in the ultrasonication bath to prevent overheating, and hence evaporation or deactivation, of the catalyst ink [12], [48], [49], [50], [51]. As an alternative, an ultrasonication probe may be employed in place of an ultrasonication bath, which delivers ultrasound energy directly to the medium via insertion of the probe [12], [50], [52]. In the lattermost case, the sample is held in an ice bath during ultrasonication to prevent potentially rapid solution overheating [50].

With these considerations in mind, four additional experiments were devised in order to evaluate the effects of power ultrasound on ionomer solutions in a setting more representative of catalyst inks: 1) Ultrasonication of 0.30 wt% sPPB-H^+^ and HMT-PMBI solutions in an ice bath (0.3 ± 0.1 °C); 2) Ultrasonication of 0.30 wt% sPPB-H^+^ and HMT-PMBI solutions containing 0.70 wt% carbon black to mimic addition of Pt/C powders; 3) Ultrasonication of 0.30 wt% sPPB-H^+^ and HMT-PMBI solutions containing 0.70 wt% carbon black, in an ice bath; and, 4) Probe ultrasonication of pure 0.30 wt% sPPB-H^+^ and HMT-PMBI solutions in an ice bath. Each series were irradiated with ultrasound for 0, 5, 10, or 20 min, after which the solutions were filtered to remove carbon (where applicable), and characterized (Table 1).

**Fig. 4:**
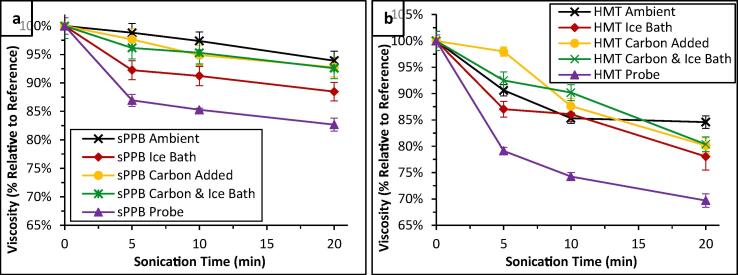
Normalized values of (a) sPPB and (b) HMT-PMBI solution viscosities (0.30 wt% in 3:1 MeOH/H_2_O) following sample ultrasonication for 0 – 20 min at ambient temperature, in an ice bath, with solutions containing added carbon black, in an ice bath with solutions containing added carbon black, and using a probe sonicator. Error bars represent the standard deviation of n = 3 unique samples.

Notable differences in polymer solution viscosities were observed (Fig. S4), which are shown as normalized values (*t_US_* = 0 min, viscosity = 100%) in Fig. 4. Surprisingly, polymer solutions ultrasonicated in an ice bath exhibited greater declines in viscosity than those ultrasonicated at ambient temperatures. This was most evident in sPPB-H^+^ polymer solutions. This effect is contradictory to expectation, because cavitation in water and methanol reaches a maximum at 35 and 19 °C, respectively, and declines at lower temperatures [28]. However, there exists a compromise between cavitation and temperature because increases in solvent temperature lead to increases in solvent vapour pressure, causing more solvent vapour to fill cavitation bubbles and effectively, paradoxically, dampening their collapse [53]. For example, D. Zhao *et. al.* found that the number of *Ganoderma lucidum* spores which were fractured (damaged) following ultrasonication in solution (DI water) increased dramatically, by nearly 20%, when experiments were performed in an ice bath versus samples processed without an ice bath [54]. It is possible that in the case of 0.15 – 1.00 wt% polymer solutions in 3:1 MeOH/H_2_O, the cumulative forces of power ultrasound within the polymer solution medium are greater at the lower temperatures (0.3 ± 0.1 °C) utilized.

It is important to consider the data in Fig. 4 with one critical caveat: reference samples (*t*_US_ = 0 min) of polymer solutions which had contained carbon black exhibited lower viscosities than samples which did not contain carbon (Fig. S4), from which it may be inferred that some polymeric material was lost due to adsorption to, and subsequent filtration of the carbon particles prior to rheological characterization. In the case of sPPB-H^+^ solutions ultrasonicated at ambient temperatures, rheology data indicated that there is no effect of carbon addition (outside of experimental error). However, when ultrasonication was conducted in an ice bath, the presence of carbon black significantly limited the ultrasound-induced decline in solution viscosity (Fig. 4a). At all experimental time intervals (*t_US_* > 0 min), samples sonicated in an ice bath *without* carbon exhibited ≥ 4.1% losses in solution viscosity compared to those which had been ultrasonicated in an ice bath *with* added carbon for the same duration of time. In the case of the anion-conducting HMT-PMBI, carbon black appeared to dampen the effects of ultrasound on solution viscosity (Fig. 4b). Solutions subjected to probe sonication exhibited the greatest rates of viscosity decline, up to 17.3 and 30.3% for sPPB-H^+^ and HMT-PMBI, respectively, after 20 min. This result was expected, because the power delivered by the ultrasonication probe (10.6 ± 0.2 W) is much higher, and more direct (i.e., does not pass through a water bath), than that of the ultrasonication bath (2.1 ± 0.1 W) used for all of the other experiments.

### 3.2 Molecular weight

**Fig. 5:**
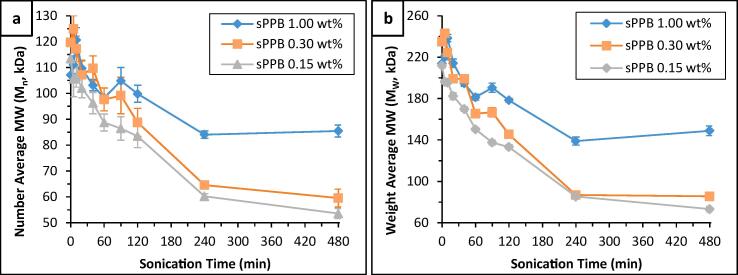
Measured molecular weights of sPPB solutions (1.00, 0.30, and 0.15 wt% in 3:1 MeOH/H_2_O) following sample bath ultrasonication for 0 – 480 min at ambient temperature: (a) *M*_n_, and (b) *M*_w_. Error bars represent the standard deviation of n = 3 unique samples.

To further evaluate the effects that bath ultrasound may have on hydrocarbon ionomer solutions, size exclusion chromatography was employed to determine their molecular weight characteristics (*M*_n_, *M*_w_, and *Đ*). Unfortunately, due to adsorption of HMT-PMBI to the size exclusion material in the SEC columns, measurements of molecular weights of HMT-PMBI were not possible. Discussions below focus only on sPPB-H^+^. A significant reduction in number average molecular weight (*M*_n_), weight average molecular weight (*M*_w_) and dispersity (*Đ*) was found for sPPB-H^+^ treated with ultrasound (Fig. 5 and S5). Lower concentration solutions (0.15 and 0.30 wt%) were more affected than higher concentration (1.00 wt%). For example, *M*_n_ was reduced by 26.4 ± 3.9% (0.15 wt%) and 25.8 ± 4.5% (0.30 wt%) in the lower concentration solution, versus 6.8 ± 3.1% (1.00 wt%) in the higher concentration solution after 120 min of ultrasound. Similarly, M_w_ was reduced by 37.1 ± 1.0% (0.15 wt%) and 38.1 ± 1.3% (0.30 wt%) in the lower concentration solutions, versus 16.6 ± 0.8% in the 1.00 wt% solution. These trends persisted through 480 min of ultrasonication. Similar to the case of polymer solution viscosity, the rate of molecular weight decrease appeared to slow with increasing sonication time.

The obtained data suggest that the ultrasonic energy causes preferential degradation of higher molecular weight polymers, hence reducing *M*_w_ at a greater rate than *M*_n_. Polymer dispersity (Fig. S5) decreased stepwise with increasing ultrasonic exposure (*t*_US_). This can be rationalized on statistical grounds: higher molecular weight polymer chains contain a disproportionately larger number of atoms which may succumb to ultrasound-induced changes, such as chain scission. In addition, it is evident that the rate of molecular weight decreases for the high concentration (1.00 wt%) sPPB-H^+^ solutions is lower than that of the medium (0.30 wt%) or low (0.15 wt%) concentration solutions. This is similar to what is observed in the case of inherent solution viscosities (Fig. 3). Interestingly, an initial *increase* in molecular weights (both *M*_n_ and *M*_w_) was observed in the 0.30 and 1.00 wt% samples following short irradiation times (*t_US_* ≤ 10 min), reaching maxima at 5 and 10 min, respectively. This effect was more pronounced with higher concentration solutions (1.00 wt%) where 12.6 ± 4.4% (M_n_) and 11.5 ± 1.6% (*M*_w_) increases were measured for solutions irradiated for 10 min. These findings correlate very well with recently published results [17], where the viscosity of Nafion® dispersions treated with ultrasound were found to increase initially (*t_US_* ≤ 10 min), predominantly in more concentrated samples, and decrease thereafter. The reasoning behind this apparent increase in molecular weight is not yet understood. It is possible that a short period of ultrasonic irradiation (*t_US_* ≤ 10 min) may promote crosslinking through *in-situ* formation of radicals [20], [21], [55]. However, it is also possible that ultrasonication increases entanglement of polymers, promoting a larger hydrodynamic radius. L. He *et. al.* [56] showed that sulfonated polyphenylene ionomers with structures similar to that of sPPB-H^+^ form bundles in dilute organic solutions (≤ 10 wt%) which persist when polymer solutions are evaporated to form membranes, partly due to their rigid backbone which prevents folding [56]. If additional clusters or bundles of polymer strands were formed within these samples, polymer solution viscosity would decrease, and following solution evaporation, their hydrodynamic radius would consequently be greater, resulting, falsely, in higher measured molecular weight values.

The SEC data unvaryingly assert that, with increasing ultrasonication, increasing polymer degradation occurs. This is likely due to the cavitation phenomenon observed in solutions subjected to ultrasound. These findings provide similar implications to the inherent viscosity data discussed above – the effects ultrasound imposes on hydrocarbon polymer solutions is reduced when the concentration of solutes is increased, because the ultrasonic energy is dissipated over a larger number of particles or macromolecules. This is an important finding, as it suggests that degradation caused by ultrasound (e.g., under ultrasonication bath operation) may be *lessened* when the matrix contains additional matter, such as Pt-supported carbon nanoparticles, as exists in the fabrication of catalyst inks.

**Fig. 6:**
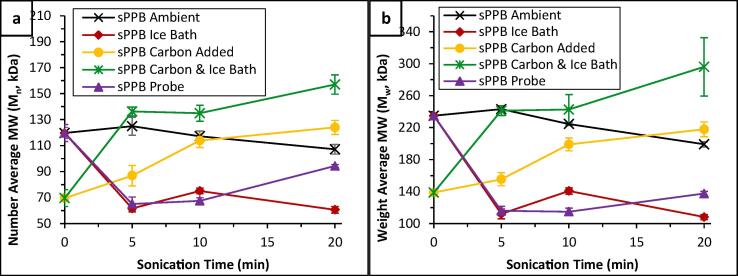
(a) number average, and (b) weight average molecular weights of sPPB solutions (0.30 wt% in 3:1 MeOH/H_2_O) following ultrasonication for 0 – 20 min at ambient temperature, in an ice bath, with solutions containing carbon black, in an ice bath with solutions containing carbon black, and using a probe ultrasonicator (26 kHz, 10.6 W). Error bars represent the standard deviation of n = 3 unique samples.

To further probe this potential effect, the change in number and weight average molecular weights of 0.30 wt% sPPB-H^+^ solutions ultrasonicated for 0–20 min, in an ice bath or in ambient conditions, in the absence or presence of added carbon black (0.70 wt%), were evaluated using size exclusion chromatography (see Fig. 6). In addition, solutions which were probe ultrasonicated were also characterized. Striking similarities were noted to the measured solution viscosities of each series of samples (Fig. 4a). When subjected to an ultrasonic bath under ambient conditions (initial temperature = 19 ± 0.1 °C), the reductions in both number average and weight average molecular weights with ultrasonication time were gradual. In contrast, ultrasonic experiments performed in an ice bath (initial temperature = 0.3 ± 0.1 °C) resulted in an immediate, dramatic decrease in polymer molecular weights. For instance, after 20 min of ultrasonication, the reductions in polymer *M*_n_ were 10.5 and 49.4%, respectively. Similar trends were observed for probe-ultrasonicated solutions, which exhibited immediate, measurable declines in polymer molecular weight and reflected the higher energy inputted by the probe (10.6 W) than that of the ultrasonication bath (2.1 W).

The addition of carbon black to the solutions which were bath ultrasonicated produced conflicting results. Solutions containing carbon which were not ultrasonicated (reference solutions) yielded polymers with greatly reduced number average (Fig. 6a) and weight average (Fig. 6b) molecular weights, suggesting that appreciable amounts of polymer adsorbed onto the added carbon black, and was subsequently removed during sample filtration prior to GPC characterization. Given these considerations, it is difficult to compare sonicated and unsonicated solutions containing carbon black. However, it remains clear that solutions containing carbon black sonicated *in an ice bath* produced a limited reduction in molecular weight upon ultrasonication. The final polymer *M*_n_ and *M*_w_ of solutions containing carbon black following 20 min of power ultrasound in an ice bath were 157.0 ± 7.5 and 296.0 ± 36.5 kDa, respectively, whereas in the case of solutions which did not contain carbon, these values were 60.6 ± 2.6 and 108.2 ± 3.3 kDa, respectively. Based upon these preliminary data, it is unclear whether presence of carbon black in a polymer solution is beneficial in mitigating ultrasound-induced polymer degradation. However, it is clear that bath ultrasonication of samples at lower temperatures has a markedly more dramatic effect than ultrasonication performed under ambient conditions.

### 3.3 ^1^H NMR spectroscopy

NMR spectroscopy is a powerful technique for characterizing organic molecules. In the case of larger macromolecules, such as hydrocarbon polymers, the complexity of NMR spectra is increased due to presence of numerous protons in slightly differing chemical environments and resolution is diminished due to restricted mobility of the chains [57]. Nevertheless, in the case of sulfonated, phenylated poly(phenylene)s (such as sPPB-H^+^) it has been shown that radical-induced degradation results in generation of an easily identifiable degradation by-product, sulfobenzoic acid (two doublets at 7.9 and 8.1 ppm in methanol‑*d*_4_, as per Fig. S6) [58].

Given that ultrasonication induces sonolysis and potential formation of reactive oxygen radical species such as the hydroxyl radical [22], [23], [27], we hypothesized that the degradation of sPPB-H^+^ may be probed using ^1^H NMR spectroscopy. If radical-induced degradation, or chemical reaction-driven chain scissions were to occur, they could be identifiable by formation of sulfobenzoic acid. When the ^1^H NMR spectra of each polymer following ultrasonication for 0 – 120 min were obtained and compared against other samples of the same concentration, no visible differences were observed, and critically, no formation of sulfobenzoic acid was detected (see Figs. S7-S9). Neither were any differences observed following rapid stirring at 1,000 rpm for 24 h (Fig. S10). However, when ultrasonication experiments were extended to 4 and 8 h, the emergence of two doublets at 7.7 and 7.9 ppm were observed in the aromatic region of the 0.15 and 0.30 wt% polymer solutions (see Figs. S11-S13). These signals coincide with previously published data on *p*-sulfobenzoic acid monopotassium salt in DMSO‑*d*_6_ [59], and may be indicative of radical-induced polymer chain scissions occurring [58].

The ^1^H NMR spectra of pure 0.30 wt% sPPB-H^+^ solutions following ultrasonication in an ice bath, and of those with added carbon black both under ambient conditions and in an ice bath are given in Figs. S14 and S15, respectively. In both cases, there were no measurable differences in the polymer spectra, as per the ultrasonic experiments conducted from 0 to 120 min above. Similarly, there were no observable differences in the spectra of sPPB-H^+^ solutions probe sonicated for 0 – 20 min (Fig. S16). This is unsurprising, because sulfonated phenylated polyphenylenes degrading via chain scission experience relatively minimal changes to bulk chemical structure, and hence negligible changes to the chemical environments of backbone protons [47], [58]. In lieu of observable degradation by-products such as sulfobenzoic acid, ^1^H NMR characterization of polymer degradation is difficult [47], [58]. Collectively, these observations suggest that sPPB-H^+^ may not degrade by sonolysis-induced free radical attack within detectable limits, but, per the stepwise reductions in both polymer molecular weight and solution viscosity observed, exhibits characteristics of degradation via chain scission as a result of acoustic cavitation.

In the case of HMT-PMBI, there is presence of distinct, non-aromatic proton chemical shifts due to both a methylated benzimidazole functional group, as well as the *hexa*-methylated *para-*terphenyl moiety in the polymer repeat unit (see Fig. 1) [8]. These protons are used to calculate the degree of methylation (ionic functionalization, Eq. S1) of the polymer backbone, which may change as result of polymer degradation [8]. In addition, the presence of upfield aliphatic protons may alleviate the difficulties typically observed in characterization of wholly-aromatic polymers [10], [57]. The degree of methylation of HMT-PMBI solutions (0.15, 0.30, and 1.00 wt%) which were not sonicated was 92.7 ± 0.5%, and hence the calculation error for degree of methylation was set to 0.5% for all subsequent experiments. Following ultrasonication for up to 480 min, no measurable differences in the degree of methylation of the polymer were calculated in any of the 0.15 and 0.30 wt% polymer solutions Fig. S17a. The highest concentration (1.00 wt%) solutions appeared to exhibit a modest decline in degree of methylation after 240 and 480 min ultrasonication – 1.0 and 1.1% lower than the reference (*t*_US_ = 0 min) sample. No trends or statistically significant variations in degree of methylation were observed in 0.30 wt% HMT-PMBI samples sonicated in an ice bath vs. under ambient conditions, with or without added carbon black, or when the probe sonicator was used (Fig. S17b).

In addition to quantitatively assessing polymer degree of methylation, each HMT-PMBI ^1^H NMR was qualitatively evaluated for changes to proton chemical shifts, as well as potential emergence of degradation by-products. In all cases, there were no differences observed in the spectra, and no evidence of formation of by-products was detected. All HMT-PMBI ^1^H NMR spectra are provided in the Supplementary Information (see Figs. S18 – S24).

### 3.4 Dosimetry measurements

**Fig. 7:**
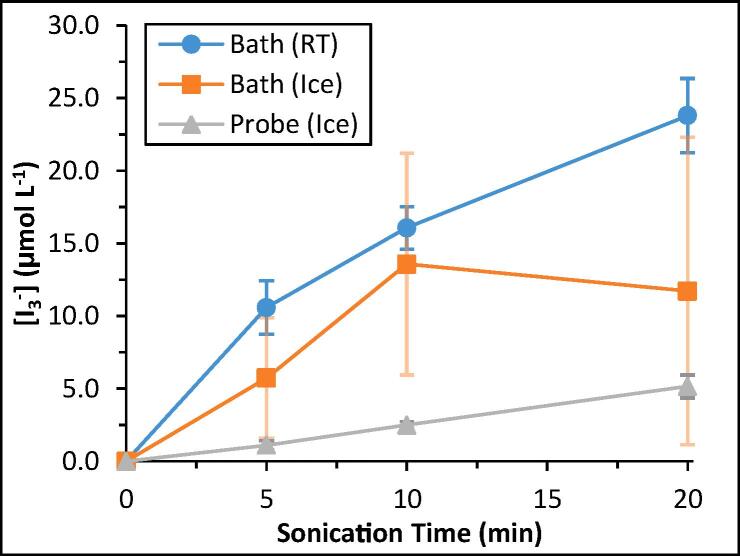
Determined concentration of I_3_^−^ anions by UV–Vis spectroscopy after 5, 10, and 20 min of bath ultrasonication at RT or in an ice bath, or via probe ultrasonication. The absorbance of I_3_^−^ was determined at 355 nm, and a molar extinction coefficient ($\varepsilon_{I_{3}^{-}})$ of 26,303 L mol^−1^*·*cm^−1^ was used to determine the concentration.[42], [43] Error bars represent the standard deviation of n = 5 unique samples.

To better compare the respective chemical environments of each ultrasonication method evaluated in this work, a series of dosimetry experiments were carried out as per the “Experimental Methods” section. By subjecting aqueous potassium iodide (KI) solutions to ultrasonication in the ultrasonication bath at RT or in an ice bath, or via direct insertion of the ultrasonication probe into a cooled solution, it was possible to trap the OH• radicals formed in each system as long-lived, UV–Vis active I_3_^−^ anions (see Eq. (4) to Eq. (7)). Fig. 7 shows the concentration of I_3_^−^ anions, [I_3_^−^], vs*.* ultrasound irradiation time under each set of conditions. Following ultrasonication for any duration, the [I_3_^−^] was in the order: probe (ice) < bath (ice) < bath (RT). For example, after 20 min of ultrasonication, the measured [I_3_^−^] was 5.2, 11.7, and 23.8 μmol·L^−1^ for solutions ultrasonicated via probe (ice), bath (ice), and bath (RT), respectively. Under the conditions employed, the sonochemical efficiency was found to be 0.011, 0.128, and 0.260 μmol·kJ^−1^ for the probe (ice), bath (ice), and bath (RT) systems, respectively, after 20 min of ultrasonication.

The slope of each line in Fig. 7 gives the rate of I_3_^−^ anion formation *ν*(I_3_^−^), which is assumed to be equal to the rate of OH• radical formation. In the case of samples subjected to probe ultrasonication (grey curve), there was a clear linear relationship between the I_3_^−^ concentration and ultrasonication time; with *ν*(I_3_^−^) equal to 0.255 μmol·L^−1^·s^−1^ (2.55 × 10^−7^ mol·L^−1^·s^−1^). The overall amount of I_3_^−^ present, however, was measurably lower than in the ultrasonication bath, despite the probe ultrasonicator operating at approximately 5 times the power of the bath (10.6 vs. 2.1 W). This suggests that the chemical environment within probe-ultrasonicated solutions, in terms of OH• radical formation, is milder than that of the ultrasonication bath. These findings are contrary to initially hypothesized results, and may be explained by the discrepancy in ultrasound frequency applied between the two methods (26 kHz ultrasound probe vs. 42 kHz ultrasound bath), because lower ultrasonic frequencies typically result in lower rates of hydroxyl radical production [60], [61], [62], [63].

The dosimetry data findings are reflected in the determined sonochemical efficiency values for each system, where the ultrasonication bath measured ≥ 11x higher than the ultrasonication probe. The cause of polymer solution viscosity and molecular weight decrease over time therefore more likely arises from mechanical forces, such as cavitation bubble implosions and acoustic streaming [64], rather than radical-induced degradation, when subject to probe ultrasonication. That is, when a liquid is ultrasonicated in the range of 20 kHz – 1 MHz, immense localized shear forces and intense agitation (especially at or near the ultrasonic transducer) occur [64]. As cavitation bubbles are formed, grow in size, and eventually violently implode, high velocity jets of liquids are generated, which can reach up to 20 m s^−1^ in velocity when in close proximity to an ultrasonic horn [64]. When occurring in the presence of macromolecules such as polymer chains, these mechanical forces may cause chain cleavage, and hence, declines in solution viscosity.

When KI solutions were ultrasonicated in the ultrasonication bath at room temperature (blue curve), and the temperature of the bath was not regulated and hence gradually increased with exposure to ultrasound energy, from 19.0 °C at 0 min, to up to 24.4 °C at 20 min. The rate of I_3_^−^ anion formation was much higher than that of the probe, and decreased as the temperature increased over time, from *ν*(I_3_^−^) = 2.12 μmol·L^−1^·s^−1^ (2.12 × 10^−6^ mol·L^−1^·s^−1^) between 0 and 5 min, to 1.10 μmol L^−1^ s^−1^ (1.10 × 10^−6^ mol·L^−1^·s^−1^) between 5 and 10 min, and 0.774 μmol·L^−1^·s^−1^ (7.74 × 10^−7^ mol·L^−1^·s^−1^) between 10 and 20 min. When the ultrasonication bath instead contained an ice bath (0.3 °C, orange curve), there was considerable variance among the measured absorbance values, with standard deviations of up to 83% in samples ultrasonicated for 20 min (n = 5). While there was measurable I_3_^−^ anion formation in all solutions examined, the variability makes it difficult to meaningfully interpret the implied rate of OH• radical formation. The irreproducibility in the ice bath sonicated samples likely arises from the presence of the ice itself, which may absorb or deflect the inputted ultrasound energy, causing appreciable deviations to the homogeneity of the ultrasound field within the water bath. Cumulatively, the dosimetry experiments confirmed the formation of hydroxyl radicals in samples ultrasonicated via both bath and probe sonication, and also suggested that the rate of hydroxyl radical formation is lower as temperatures increase.

### 3.5 Effect of ultrasonicated catalyst inks on fuel cell performance

A series of *in-situ* fuel cell analyses using sPPB-H^+^, and Nafion® D520 as a PFSA reference material, were performed to evaluate the effects of ultrasonication on ionomers in a catalyst ink. In order to separate the process of ink fabrication from ultrasonication of the polymer solutions, each polymer solution was subjected to ultrasound prior to its addition to, and use, in the catalyst ink. That is, 1.00 wt% solutions of sPPB-H^+^ and Nafion® D520 (diluted) were either untreated (reference samples), bath ultrasonicated for 20 min (rt), or probe ultrasonicated for 20 min (ice bath) *prior* to their use as ionomer in catalyst inks. Each catalyst ink was subsequently prepared using the same methodology, as described in the Experimental Methods section and Supplementary Information, then integrated into a membrane electrode assembly (MEA) employing a Nafion® 211 membrane, and evaluated in a hydrogen/oxygen fuel cell.

**Fig. 8:**
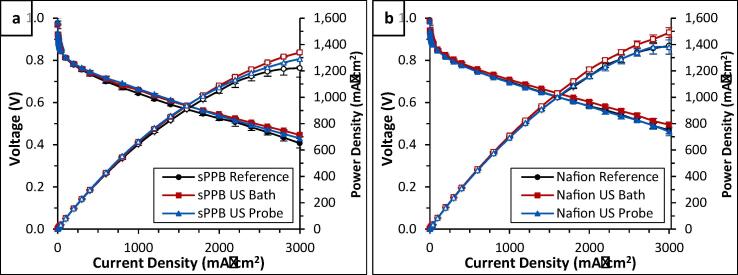
Polarization (left y-axis) and power density (right y-axis) plots of MEAs prepared incorporating either (a) sPPB-H^+^ (ultrasonicated - bath and probe - and unsonicated) or (b) Nafion® (ultrasonicated - bath and probe - and unsonicated) as ionomer (binder) in the catalyst layer. Fuel cell polarization data were obtained at 80 °C, H_2_ anode and O_2_ cathode, 100% RH, 1 atm pressure. Error bars represent the standard deviation of n = 3 independently fabricated and assessed MEAs.

Averaged (n = 3) polarization (left axis) and power density (right axis) plots for each series of MEAs are shown in Fig. 8. A peak power density of 1,338 ± 26 mW·cm^−2^ was measured for MEAs incorporating pre-sonicated sPPB-H^+^ ionomer via the ultrasonication bath (US Bath, Fig. 8a). This cell performance is similar to MEAs incorporating the probe pre-sonicated sPPB-H^+^ solution (US Probe, 1,290 ± 30 mW·cm^−2^), and to the reference (unsonicated) sPPB-H^+^ solution (1,220 ± 40 mW·cm^−2^). There is evidence to suggest that MEAs prepared with pre-sonicated sPPB-H + ionomer provide a marginally higher current density in the very high current regime, but these regions of voltage are of lower significance in fuel cell devices, and even so, the differences are minimal and may well fall within experimental error. Similar observations are observed in fuel cells incorporating Nafion® ionomer, as shown in Fig. 8b. Nafion® solutions which were pre-sonicated via ultrasonication bath yielded MEAs exhibiting a maximum power density of 1,490 ± 37 mW·cm^−2^. The maximum power density of cells incorporating probe ultrasonicated Nafion® solution (1,390 ± 55 mW·cm^−2^) were similar to that of the reference (unsonicated) Nafion® solution (1,380 ± 55 mW·cm^−2^). The low current density regions (activation region, < 100 mA·cm^−2^) of these plots are provided in the Supporting Information (Fig. S28a and b, respectively). In this region, there were no differences in cell performance outside of experimental error.

Statistical significance of these data was examined using 2-tailed *T*-test analyses performed to 95% confidence intervals; results are provided in the Supplementary Information (Tables S1 – S6). These analyses showed that sPPB-H^+^ US samples (sPPB US Bath and sPPB US Horn) exhibited insignificant differences in performance in low voltage regions versus the reference sample (sPPB Reference), and that the two different ultrasonication methods were not statistically different from one another. That is, a pre-sonicated sPPB-H^+^ ionomer resulted in MEAs which exhibited similar performances than that of the reference, and the method of sonication used (US Bath or US Horn) did not have a significant effect (Fig. 8a). In the case of Nafion®-containing MEAs, the Nafion® US Bath performance curve was no different than both the Nafion® US Probe and Nafion® reference performance curves in the lower voltage regions.

To further probe the observed fuel cell performance, the electrochemically active surface area (ECSA, see Fig. S25), charge transfer resistance (*R*_ct_, see Fig. S26), and ionic resistance (*R*_Ionic_, see Fig. S27) of the catalyst layer of each MEA were obtained using previously described methodology [45]. Data are provided in Table 3. In both cases (sPPB-H^+^ vs. Nafion®), no significant differences in ECSA were found upon ultrasonicating the ionomer. One of the reasons ultrasonication of catalyst inks is necessary is because the catalyst ink must be uniformly dispersed on the substrate, free of agglomerates and inconsistencies [50]. We originally speculated that ultrasonication of polymer solutions in the ultrasonication bath prior to their integration into catalyst inks may impose changes in polymer agglomeration and entanglement, which enables better dispersion of the resulting catalyst ink. Previously, M. Wang *et. al.*, [50] reported a measurable difference in fuel cell catalyst layer ECSA when using either a probe or bath ultrasonicator, for 1 to 20 min. For example, a higher ECSA was obtained in catalyst layers prepared via probe ultrasonication for 1 min, versus 20 min, but conversely, the ECSA was lower in catalyst layers prepared using an ultrasonication bath for 1 min, versus 20 min [50].

**Table 3:** Measured and calculated values for catalyst layer electrochemically active surface area (ECSA), charge transfer resistance (*R*_ct_), and ionic resistance (*R*_ionic_) for catalyst layers of sPPB-H^+^ and Nafion®-containing MEAs. Error is reported as the standard deviation of n = 3 independently fabricated and assessed MEAs.

Polymer Solution	ECSA (m^2^.g^−1^_Pt_)	*R*_ionic_ (mΩ.cm^−2^)	*R*_ct_ (mΩ.cm^−2^)
sPPB-H^+^ Reference	39.6 ± 4.7	34.7 ± 5.8	903 ± 61
sPPB-H^+^ US Bath	45.0 ± 5.7	52.2 ± 6.8	822 ± 54
sPPB-H^+^ US Probe	36.6 ± 9.4	54.9 ± 9.2	830 ± 48
Nafion® Reference	52.2 ± 6.9	44.0 ± 8.5	415 ± 42
Nafion® US Bath	56.7 ± 4.6	39.3 ± 7.6	407 ± 28
Nafion® US Probe	45.1 ± 5.9	33.3 ± 6.9	414 ± 38

The ionic resistance (*R*_ionic_) of sPPB-H^+^ containing catalyst layers decreased in the order of US Probe ≈ US Bath > reference solution. That is, pre-sonicating sPPB-H^+^ solutions using either the ultrasonication bath or probe yielded catalyst layers with marginally higher resistance to protonic transport, which is disfavourable. Conversely, the ionic resistance of Nafion®-containing catalyst layers were all within experimental error. In both cases, it is evident that measurable changes occurred to the ionomer in solution when it was sonicated prior to its integration into catalyst layers. The increases in ionic resistance of sPPB-H^+^-containing MEAs may be due to changes in polymer dispersion, solution phase macrostructures, or degradation, leading to disfavored blockages of mesoporous proton transport channels within the catalyst layer. The charge transfer resistance of both the sPPB-H^+^, and Nafion®-containing catalyst layers were experimentally indistinguishable (within error; statistically identical via *T-test* analyses as per Table S7).

## 4 Conclusions

A systematic study was performed to investigate the impact of low-frequency, high-power ultrasound on two different hydrocarbon-based ionomers: proton exchange polymer sPPB-H^+^, and anion exchange polymer HMT-PMBI. Ionomer solutions (1.00, 0.30, and 0.15 wt%) in 3:1 methanol/water (v/v) were subject to ultrasound irradiation in either a laboratory ultrasonication bath at room temperature or in an ice bath, or, via direct insertion of an ultrasonication probe into a cooled solution for various durations between 0 and 480 min. The effects of ultrasound were assessed by measuring polymer solution viscosity (*η*), molecular weight parameters (*M*_n_, *M*_w_, and *Đ*), and chemical structure (via ^1^H NMR) of irradiated samples in comparison to their respective reference samples. In addition, catalyst inks formulated using either non–, bath-, or probe-sonicated sPPB-H^+^, and Nafion® D520 as a PFSA reference, were used to prepare MEAs for *in-situ* PEM fuel cell measurements.

Following ultrasonication, stepwise reductions in polymer solution viscosity were noted in both sPPB-H^+^ (up to 25%) and HMT-PMBI (up to 43%). The largest effect was observed in lower concentration polymer solutions, which we speculate is due to fewer macromolecules being present in the solution to dissipate the incoming ultrasound irradiation. Employing an ice bath or using an ultrasonication probe resulted in a greater reduction in polymer solution viscosity upon sonication. These findings were corroborated by a similar decrease in molecular weight of sPPB-H^+^ with ultrasonication time, where striking similarities to the decreases in solution viscosity were noted. Polymer molecular weights decreased more rapidly when ultrasonication was performed on solutions of low concentration, and at colder temperatures (ice bath), but were mitigated when the sonicated polymer solutions contained carbon black in amounts similar to that used in fuel cell catalyst inks. The data revealed that, with increasing ultrasonication, there is increasing polymer degradation, most likely due to solution cavitation induced by power ultrasound.

^1^H NMR analysis revealed only trace formation of sulfobenzoic acid, a radical-induced degradation product of sPPB-H^+^, after 240 min of bath ultrasonication. Conversely, no significant differences were observed in the ^1^H NMR spectra of HMT-PMBI samples following any solution ultrasonication. Dosimetry experiments performed to approximate the rate of HO• radical formation under each ultrasonication condition revealed that bath ultrasonication at room temperature (1.190 mol·L^−1^·s^−1^) or bath ultrasonication in an ice bath (0.585 mol·L^−1^·s^−1^) produced higher rates of HO• radical formation than probe ultrasonicated (0.255 mol·L^−1^·s^−1^), despite the bath sonicator operating at a lower inputted power (2.1 vs 10.6 W, respectively). These data indicated that both sPPB-H^+^ and HMT-PMBI do not appreciably degrade by sonolysis-induced free radical attack, within detectable limits, but rather through mechanical chain scission as a result of ultrasound-induced solution cavitation.

*In-situ* fuel cell evaluation of pre-sonicated sPPB-H^+^, as well as Nafion® D520 as a PFSA reference material, revealed that sonication of the polymer solutions prior to their use as ionomer in the catalyst layer yielded insignificant differences in the voltage regime where fuel cells are usually operated (0.8–0.6 V). The results reveal the finding that ultrasound of ionomer solutions, while leading to chain scission of polymer chains, has little impact on the performance of MEAs prepared therefrom. We recognize that, while the fuel cell data reported here are reproducible, clearly there are many different, interdependent facets that lead to a particular fuel cell performance. These preliminary studies address questions concerning the role of ultrasound on ionomers in the preparation of catalyst inks and provide the motivation for further detailed studies on their effect on catalyst layers and fuel cell dynamics.

## Declaration of Competing Interest

The authors declare that they have no known competing financial interests or personal relationships that could have appeared to influence the work reported in this paper.

## Footnotes

Appendix A Supplementary data to this article can be found online at https://doi.org/10.1016/j.ultsonch.2021.105588.
