# Supplementary material for: Does power ultrasound affect hydrocarbon Ionomers?
Source: Ultrason Sonochem. 2021 May 7;75:105588. doi: 10.1016/j.ultsonch.2021.105588 (PMC8141775; doi:10.1016/j.ultsonch.2021.105588)
Supplement: Supplementary data 1 [file mmc1.pdf]

Supporting Information for

Does Power Ultrasound affect Hydrocarbon Ionomers?

Michael Adamski†1, Nicolas Peressin†1, Emmanuel Balogun[1], Bruno G. Pollet[2] and Steven Holdcroft*1

[1]Holdcroft Research Group, Department of Chemistry, Simon Fraser University, 8888 University Drive, Burnaby BC, V5A 1S6, Canada

[2]Hydrogen Energy and Sonochemistry Research Group, Department of Energy and Process Engineering, Norwegian University of Science and Technology (NTNU), NO-7491, Trondheim, Norway

*To whom correspondence should be addressed:

holdcrof@sfu.ca

†These authors contributed equally to this work.

Table of Contents

List of Tables 2

List of Figures 3

Experimental Methods 5

Rheology Data 7

GPC Data 10

^{1}H NMR Data 11

***In-situ* Characterization Data** 28

***T-Test* Statistically Significant Analyses** 32

References 39

List of Tables

Table S1. Nafion polarization curves T-test – Reference versus US Bath. T-tests were performed for each data point to 95% confidence limits, where $p < 0.05$ indicates statistically different values........ **32**

Table S2. Nafion polarization curves T-test – Reference versus US Probe. T-tests were performed for each data point to 95% confidence limits, where $p < 0.05$ indicates statistically different values........ **33**

Table S3. Nafion polarization curves T-test – US Bath versus US Probe. T-tests were performed for each data point to 95% confidence limits, where $p < 0.05$ indicates statistically different values........ **34**

Table S4. sPPB-H^+ polarization curves T-test – Reference versus US Bath. T-tests were performed for each data point to 95% confidence limits, where $p < 0.05$ indicates statistically different values........ **35**

Table S5. sPPB-H^+ polarization curves T-test – Reference versus US Probe. T-tests were performed for each data point to 95% confidence limits, where $p < 0.05$ indicates statistically different values........ **36**

Table S6. sPPB-H^+ polarization curves T-test – US Bath versus US Probe. T-tests were performed for each data point to 95% confidence limits, where $p < 0.05$ indicates statistically different values........ **37**

Table S7. T-tests for sPPB-H+ reference, US bath, and US probe charge transfer resistance. T-tests were performed to 95% confidence limits, where $p < 0.05$ indicates statistically different values........**38**

List of Figures

Figure S1. Measured average viscosities (mPa·s) of (a) sPPB-H^+ and (b) HMT-PMBI samples sonicated for 0 – 480 min. **7**
Figure S2. Normalized average viscosities (%) of (a) 1.00 wt%, (b) 0.30 wt%, (c) 0.15 wt% sPPB-H^+ and (d) 1.00 wt%, (e) 0.30 wt%, (f) 0.15 wt% HMT-PMBI samples sonicated for 0 – 480 min, including samples subject to rapid stirring (1,000 rpm) for 24 h (circle markers). **8**
Figure S3. Normalized values of calculated inherent viscosities of (a) sPPB and (b) HMT-PMBI solutions (1.00, 0.30, and 0.15 wt% in 3:1 MeOH/H2O) following sample ultrasonication for 0 – 480 min.......... **9**
Figure S4. Measured average viscosities (mPa·s) of (a) sPPB-H^+ and (b) HMT-PMBI solutions (0.30 wt% in 3:1 MeOH/H_2O) following sample ultrasonication for 0 – 20 min at ambient temperature, in an ice bath, with solutions containing added carbon, in an ice bath with solutions containing added carbon, and using a probe sonicator........ **9**
Figure S5. Measured dispersities, Đ (M_w/M_n) of sPPB solutions (1.00, 0.30, and 0.15 wt% in 3:1 MeOH/H_2O) following sample ultrasonication for 0 – 480 min. **10**
Figure S6. Aromatic region of a sulfonated phenylated polyphenylene ^{1}H NMR spectra (in methanol-d_4) before and after hydroxyl radical-induced degradation (0.30% H_2O_2 in H_2O solution for 100 h at 80 °C), showing formation of the major sulfobenzoic acid degradation by-product.[60] **11**
Figure S7. ^{1}H NMR spectra of sPPB-H^+ (0.15 wt% solutions) following ultrasound treatments from 0 – 120 min. **11**
Figure S8. ^{1}H NMR spectra of sPPB-H^+ (0.30 wt% solutions) following ultrasound treatments from 0 – 120 min. **12**
Figure S9. ^{1}H NMR spectra of sPPB-H^+ (1.00 wt% solutions) following ultrasound treatments from 0 – 120 min. **13**
Figure S10. ^{1}H NMR spectra of sPPB-H^+ (all solutions) following rapid stirring (1,000 rpm, 24 h). **14**
Figure S11. Aromatic regions of 0.15 wt% sPPB-H^+ solution ^{1}H NMR spectra following 0, 240, and 480 min ultrasonication. **15**
Figure S12. Aromatic regions of 0.30 wt% sPPB-H^+ solution ^{1}H NMR spectra following 0, 240, and 480 min ultrasonication. **16**
Figure S13. Aromatic regions of 1.00 wt% sPPB-H^+ solution ^{1}H NMR spectra following 0, 240, and 480 min ultrasonication. **17**
Figure S14. ^{1}H NMR spectra of sPPB-H^+ (0.30 wt% solutions) following ultrasonication for 0 – 20 min in an ice bath. The additional upfield proton signals between 3.39 and 2.69 ppm found in 5, 10, and 20 min samples are due to impurities from NMR sample vial caps, because PTFE caps were unavailable due to COVID-19-associated supply chain shortages. **18**
Figure S15. ^{1}H NMR spectra of sPPB-H^+ (0.30 wt% solutions) following ultrasonication for 0 – 20 min with added carbon black under ambient conditions and in an ice bath. The additional upfield proton signals between 3.50 and 2.69 ppm found in all samples are due to impurities from NMR sample vial caps, because PTFE caps were unavailable due to COVID-19-associated supply chain shortages. **19**
Figure S16. ^{1}H NMR spectra of sPPB-H^+ (0.30 wt% solutions) following probe ultrasonication for 0 – 20 min. The additional upfield proton signals between 3.39 and 2.69 ppm found in 5, 10, and 20 min

samples are due to impurities from NMR sample vial caps, because PTFE caps were unavailable due to COVID-19-associated supply chain shortages.. 20
Figure S17. Calculated degree of methylation for HMT-PMBI polymer solutions subject to (a) ultrasonication for 0 – 480 min (0.15, 0.30, and 1.00 wt% in 3:1 MeOH/H_2O), and (b) 0.30 wt% in 3:1 MeOH/H_2O solutions following sample ultrasonication for 0 – 20 min at ambient temperature, in an ice bath, with solutions containing added carbon black, in an ice bath with solutions containing added carbon black, and using a probe sonicator (26 kHz, 10.6 W). 20
Figure S18. ^{1}H NMR spectra of HMT-PMBI (0.15 wt% solutions) following ultrasound treatments from 0 – 480 min... 21
Figure S19. ^{1}H NMR spectra of HMT-PMBI (0.30 wt% solutions) following ultrasound treatments from 0 – 480 min... 22
Figure S20. ^{1}H NMR spectra of HMT-PMBI (1.00 wt% solutions) following ultrasound treatments from 0 – 480 min... 23
Figure S21. ^{1}H NMR spectra of HMT-PMBI (all solutions) following rapid stirring (1,000 rpm, 24 h). .. 24
Figure S22. ^{1}H NMR spectra of HMT-PMBI (0.30 wt% solutions) following ultrasonication for 0 – 20 min in an ice bath. The additional upfield proton signals between 3.39 and 2.69 ppm found in 5, 10, and 20 min samples are due to impurities from NMR sample vial caps, because PTFE caps were unavailable due to COVID-19-associated supply chain shortages.. 25
Figure S23. ^{1}H NMR spectra of HMT-PMBI (0.30 wt% solutions) following ultrasonication for 0 – 20 min with added carbon black under ambient conditions and in an ice bath. The additional upfield proton signals between 3.50 and 2.69 ppm found in all samples are due to impurities from NMR sample vial caps, because PTFE caps were unavailable due to COVID-19-associated supply chain shortages.. 26
Figure S24. ^{1}H NMR spectra of HMT-PMBI (0.30 wt% solutions) following probe ultrasonication for 0 – 20 min. 27
Figure S25. ECSA data extracted from the CV analysis of MEAs containing Nafion® (blue) or sPPB-H^+ (red) ionomer in the catalyst layer. Polymer solutions used in MEA preparation were pre-treated for 20 min with either an ultrasonication bath (US Bath) or probe (US Probe), or left untreated (reference). CV scans were performed at 80 °C, H_2 anode and N_2 cathode, 100% RH, 1 atm pressure.28
Figure S26. Nyquist plots illustrating the high-frequency intercept and low-frequency intercept by a linear fit of low-frequency data, used to determine catalyst layer ionic resistance of MEAs containing (a) sPPB-H^+, or (b) Nafion® ionomer in the catalyst layer. Polymer solutions used in MEA preparation were pre-treated for 20 min with either an ultrasonication bath (US Bath) or probe (US Probe), or left untreated (reference). Characterization conditions were 80°C, H_2 anode and N_2 cathode gases, 100% RH, 1 atm pressure.. 29
Figure S27. Nyquist plots of MEAs containing (a) sPPB-H^+, or (b) Nafion® ionomer in the catalyst layer. Polymer solutions used in MEA preparation were pre-treated for 20 min with either an ultrasonication bath (US Bath) or probe (US Probe), or left untreated (reference). EIS spectra recorded were at 0.8 V, 80°C, H_2 anode and O_2 cathode, 100% RH, 1 atm pressure, and data was used to calculate charge transfer resistance, R_{ct}. 30

Experimental Methods

SEC and ^{1}H NMR

Size exclusion chromatography measurements were collected via a triple detection technique using a combination of refractive index, right angle light scattering, and viscometer detectors. Narrow molecular weight distribution polystyrene standards (PS; M_w = 105,982, M_n = 101,335 g·mol^{-1}) were used to calibrate the system. Calibration was verified by measuring a standard with a wider dispersity (PS; M_w = 247,581, M_n = 104,485 g·mol^{-1}). Polymer samples were prepared to 5.0 ± 0.1 mg·mL^{-1} concentrations in HPLC grade DMF (containing 0.01 M LiBr) and filtered through a 0.22 µm, 13 mm diameter CELLTREAT® PTFE filter. The injection volume was 100 µL with a flow rate of 1.0 mL·min^{-1}, and the column and detector temperatures were held constant at 50 °C throughout the measurement. GPC analyses were performed thrice on each sPPB-H$^+$ polymer sample to obtain average molecular weight and dispersity values, and measurement error, which is reported as the standard deviation.

The ^{1}H spectra residual solvent peak for DMSO-d_6 was set to 2.50 ppm. To calculate degree of methylation (dm) of HMT-PMBI, the ^{1}H NMR spectrum was acquired and a baseline correction (MestReNova, "Full Auto Polynomial Fit") was applied, after which the integration region of 4.30-3.78 ppm (12.00 H) was compared against the integration region of 3.78-3.55 ppm (x H).[1] The dm% was then calculated per Equation S1.[1]

$$dm\% = 50 \times \left(\frac{1}{1+\left(\frac{x}{6}\right)}\right) + 50 \qquad \text{(Equation S1)}$$

Dosimetry

0.10 mol L^{-1} KI solutions were partitioned into 27.0 ± 0.1 mL samples contained within 30 mL, 9 cm tall VWR® glass vials. The resulting samples were subject to individual experiments utilizing either an ultrasound bath at room temperature, ultrasound bath with ice, or probe sonicator with ice, for 0, 5, 10, and 20 minutes. Experiments at each time interval were repeated at minimum three times, with the error being reported as the standard deviation. Spectroscopy measurements were performed using a Perkin Elmer Lambda 850 UV-Vis Spectrometer, and samples were measured in 3.5 mL quartz cuvettes with path lengths of 1.0 cm. Samples were measured against deionized water as a blank. Non-sonicated (0 minute) samples contained no triiodide anion (0 mol·L^{-1} I_3^-), and therefore, the measured absorbance of the non-sonicated (0 minute) KI samples were subtracted from the absorbance of all reported samples.

Membrane Electrode Assembly and Fuel Cell Operation

To prepare a catalyst ink, a given ionomer solution (US-Bath, US-Probe, or reference) was mixed appropriately with the corresponding quantity of Pt/C catalyst and solvents (methanol and de-ionized H_2O) to a final target of 0.30 wt% Nafion® ionomer and 0.70 wt% inorganic solids,[3] or 0.15 wt% sPPB-H$^+$ and 0.85 wt% inorganic solids.[4]. A third of the required water was added initially to the Pt/C solid, and the resulting slurry solution was ultrasonicated for 10 min to ensure the Pt/C particles were well

wetted. The slurry solution was placed on a stir plate (1,000 rpm) in a fume hood, and a third of the required methanol was added dropwise and stirred for 15 min. To the resulting dispersion was added ionomer solution dropwise, and the resulting mixture was stirred for 10 min. The remaining quantity of methanol and water were then added dropwise, sequentially. The obtained catalyst ink solution was then ultrasonicated for 2 hours in an ultrasonication bath filled with ice water.

Catalyst inks were spray-coated onto Nafion® NR-N211 membranes (DuPont, TE143904) using an automatic spray coater (Sono-tek ExactaCoat). The Pt loading on both anode and cathode electrodes was 0.4 mg Pt cm^{-2}. The resulting catalyst coated membranes (CCM) were sandwiched between two 5 cm^2 (2.24 x 2.24 cm) PTFE treated gas diffusion layers (Sigracet 29BC, FuelCellStore) and compressed in fuel cell hardware (AHNS Co.) using a torque wrench, with the adequacy of compression determined by pressure-sensitive paper (Fujifilm).[5–7] Compression evaluations were first conducted on our hardware, consisting of eight bolts evenly spaced bolts, at different applied torque values from 0.5 to 6.0 Nm with a maximum increment of 0.5 Nm. Preferred compression was obtained at 5.7 Nm, which is equivalent to 50 in-lbs, wherein desirable cell pressure distributions and repeatable cell performance were obtained. Three identical membrane electrode assemblies (MEAs) were prepared for each of the aforementioned ultrasonic bath (US bath), ultrasonic probe (US probe), and reference catalyst inks. That is, a total of 9 MEAs containing Nafion® ionomer in the catalyst layers, and 9 MEAs containing sPPB-H^+ ionomer in the catalyst layers (total 18 MEAs) were evaluated. This number of samples was evaluated to assure reproducibility of results and verify the accuracy of reported data. The cell hardware was connected to a fuel cell test station (Scribner 890CL). All errors reported are the corresponding standard deviations.

MEAs were conditioned using the previously reported cathode starvation procedure.[8] The fuel cell temperature was set at 80 °C and 100% relative humidity (RH) at both electrodes. The inlet gas flows were 0.5 slpm hydrogen at the anode and 1.0 slpm oxygen at the cathode. The electrochemical characteristics of the MEAs were investigated using polarization data, electrochemical impedance spectroscopy (EIS) and cyclic voltammetry (CV). The electrochemical characterization techniques employed in this work have been previously detailed in a report by E. Balogun, *et. al*.[8] Electrochemical impedance spectroscopy was used to determine charge transfer resistance of the fuel cell under H_2/O_2 (anode/cathode) operation,[9] and the ionic resistance of the catalyst layer under H_2/N_2 (anode/cathode) operation.[10]

To determine the ionic resistance of the catalyst layer, a double parallel resistive rail was used, as detailed by Z. Qi *et. al.*,[10] and M. Lefebvre *et. al.*[9] One of the parallel resistive rails represents the electron transport through the conducting carbon support, and the other represents ion transport through the inter-particle regions corresponding to ion migration through the catalyst layer. The low-frequency resistance (LFR) is represented by an upward curve in the impedance plot, to a limiting capacitive response (vertical) which corresponds to the total capacitance and resistance of the catalyst layer. The ionic resistance, R_{ionic}, was obtained from the length of the Warburg-like region projected onto the real impedance (Z′) axis ($R_{ionic}/3$). The membrane's ionic resistance was obtained from the

high-frequency intercept (HFR) with the x-axis on the Nyquist plot.[11] The electrode's ionic resistance ($R_{E\cdot ionic}$) was calculated using Equation S2, where LFR is the low frequency resistance.

$$LFR = \frac{R_{E\cdot ionic}}{3} \qquad \text{(Equation S2)}$$

Measurement were performed using a VersaStat 4 potentiostat, with H_2 and N_2 gas flows at the anode and cathode, respectively, each set to 0.5 standard litre per minute (SLPM). Potentiostatic EIS measurements were performed with a start frequency of 1 Hz and end frequency of 100 kHz, with a root mean squared amplitude of 10 mV and potential step of 0.45 V.

Rheology Data

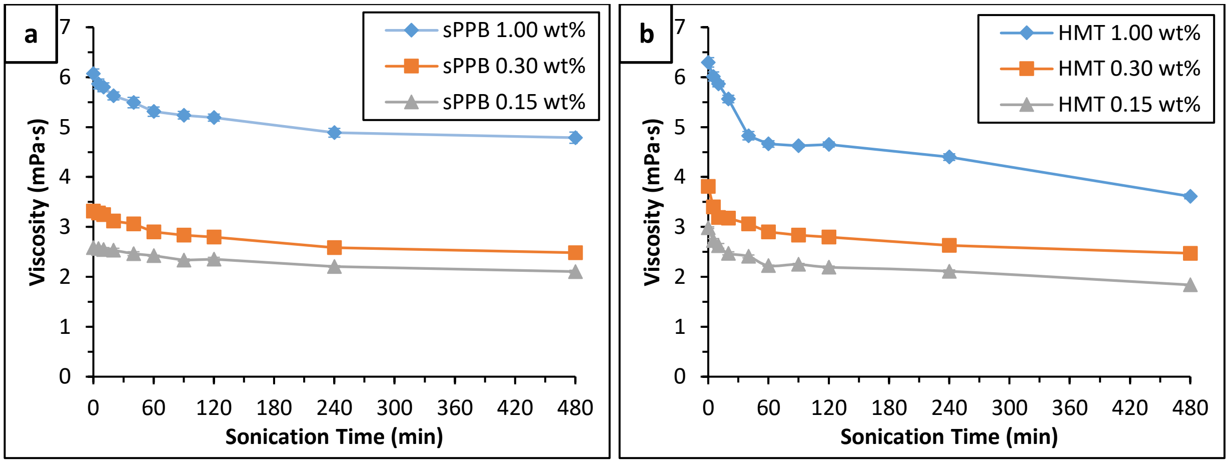

Figure S1. Measured average viscosities (mPa·s) of (a) sPPB-H^+ and (b) HMT-PMBI samples sonicated for 0 – 480 min. Error bars represent the standard deviation of n = 3 unique samples.

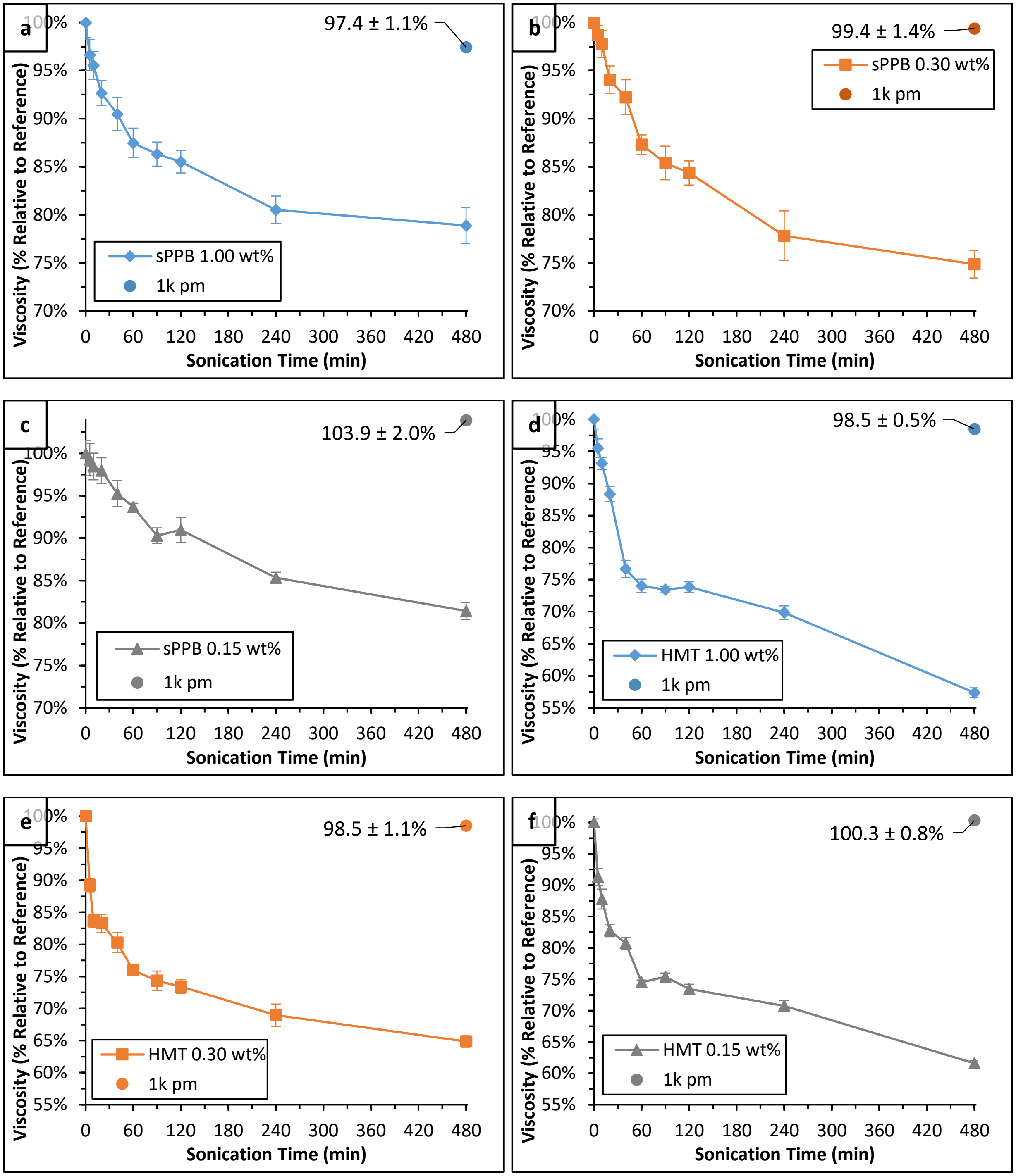

Figure S2. Normalized average viscosities (%) of (a) 1.00 wt%, (b) 0.30 wt%, (c) 0.15 wt% sPPB-H^+ and (d) 1.00 wt%, (e) 0.30 wt%, (f) 0.15 wt% HMT-PMBI samples sonicated for 0 – 480 min, including samples subject to rapid stirring (1,000 rpm) for 24 h (circle markers). Error bars represent the standard deviation of n = 3 unique samples.

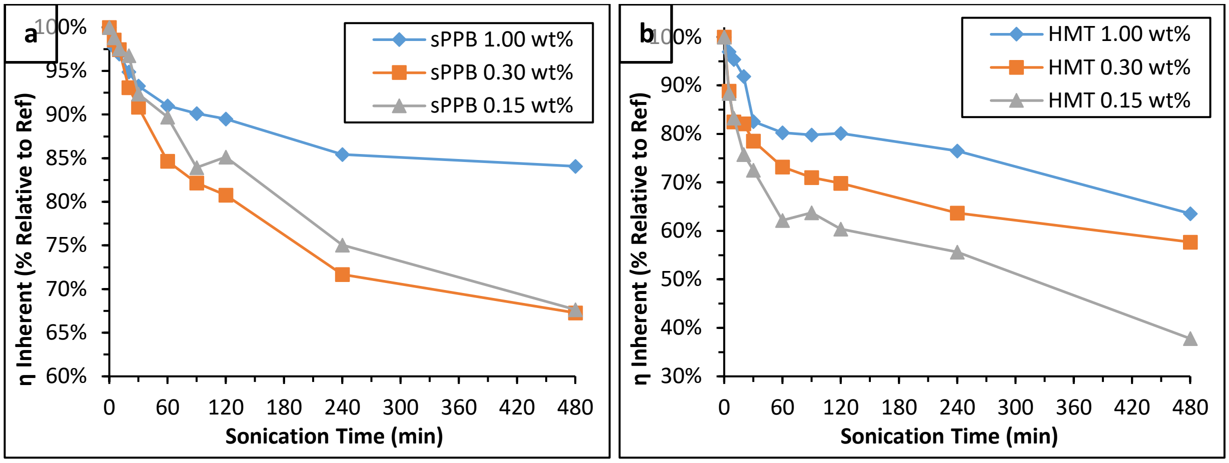

Figure S3. Normalized values of calculated inherent viscosities of (a) sPPB and (b) HMT-PMBI solutions (1.00, 0.30, and 0.15 wt% in 3:1 MeOH/H2O) following sample ultrasonication for 0 – 480 min. Error bars represent the standard deviation of n = 3 unique samples.

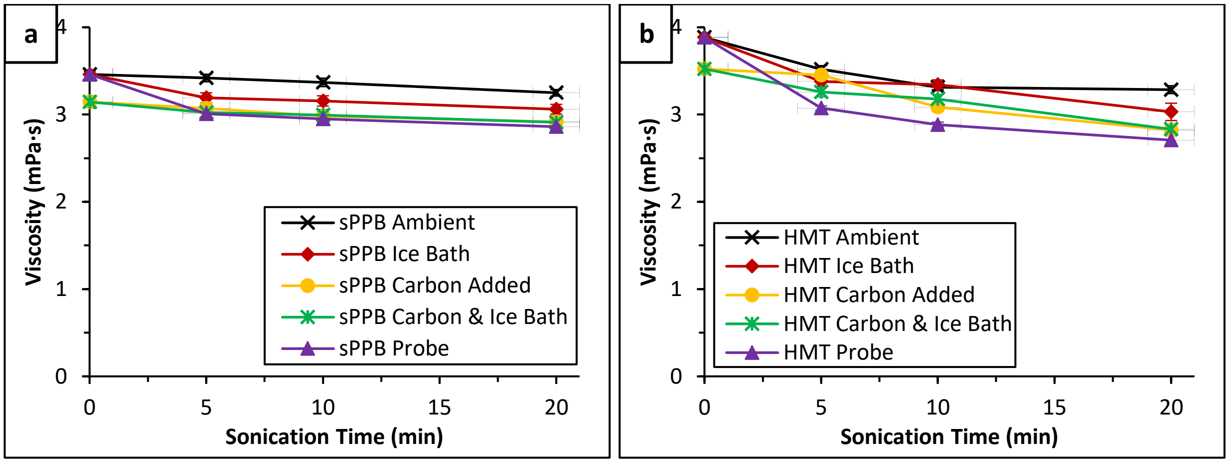

Figure S4. Measured average viscosities (mPa·s) of (a) sPPB-H^+ and (b) HMT-PMBI solutions (0.30 wt% in 3:1 MeOH/H_2O) following sample ultrasonication for 0 – 20 min at ambient temperature, in an ice bath, with solutions containing added carbon, in an ice bath with solutions containing added carbon, and using a probe sonicator. Error bars represent the standard deviation of n = 3 unique samples.

GPC Data

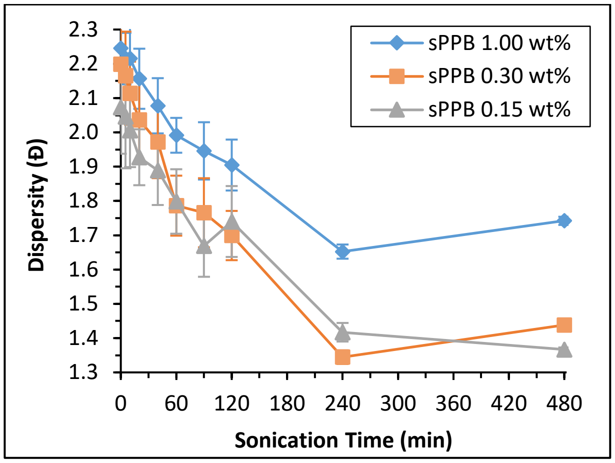

Figure S5. Measured dispersity, Đ (M_w/M_n) of sPPB solutions (1.00, 0.30, and 0.15 wt% in 3:1 MeOH/H_2O) following sample ultrasonication for 0 – 480 min. Error bars represent the standard deviation of n = 3 unique samples.

^{1}H NMR Data

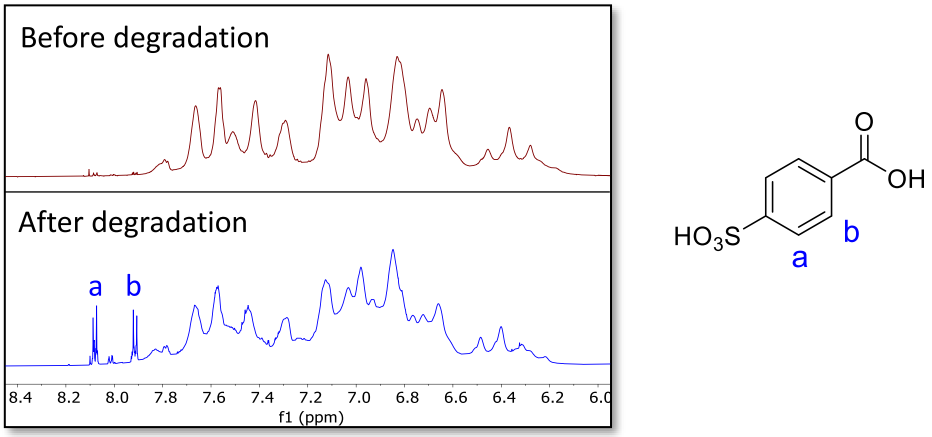

Figure S6. Aromatic region of a sulfonated phenylated polyphenylene ^{1}H NMR spectra (in methanol-d_4) before and after hydroxyl radical-induced degradation (0.30% H_2O_2 in H_2O solution for 100 h at 80 °C), showing formation of the major sulfobenzoic acid degradation by-product.[12]

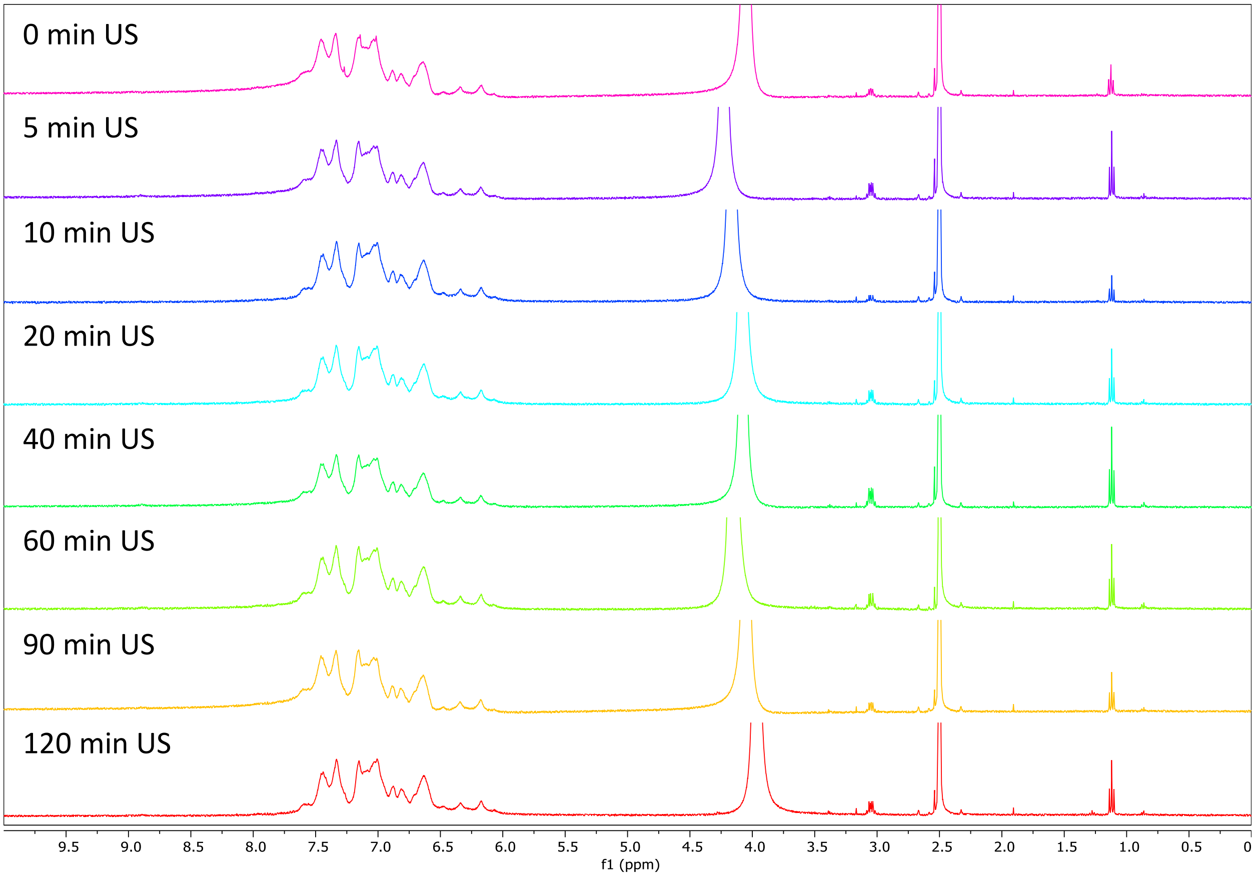

Figure S7. ^{1}H NMR spectra of sPPB-H$^+$ (0.15 wt% solutions) following ultrasound treatments from 0 – 120 min.

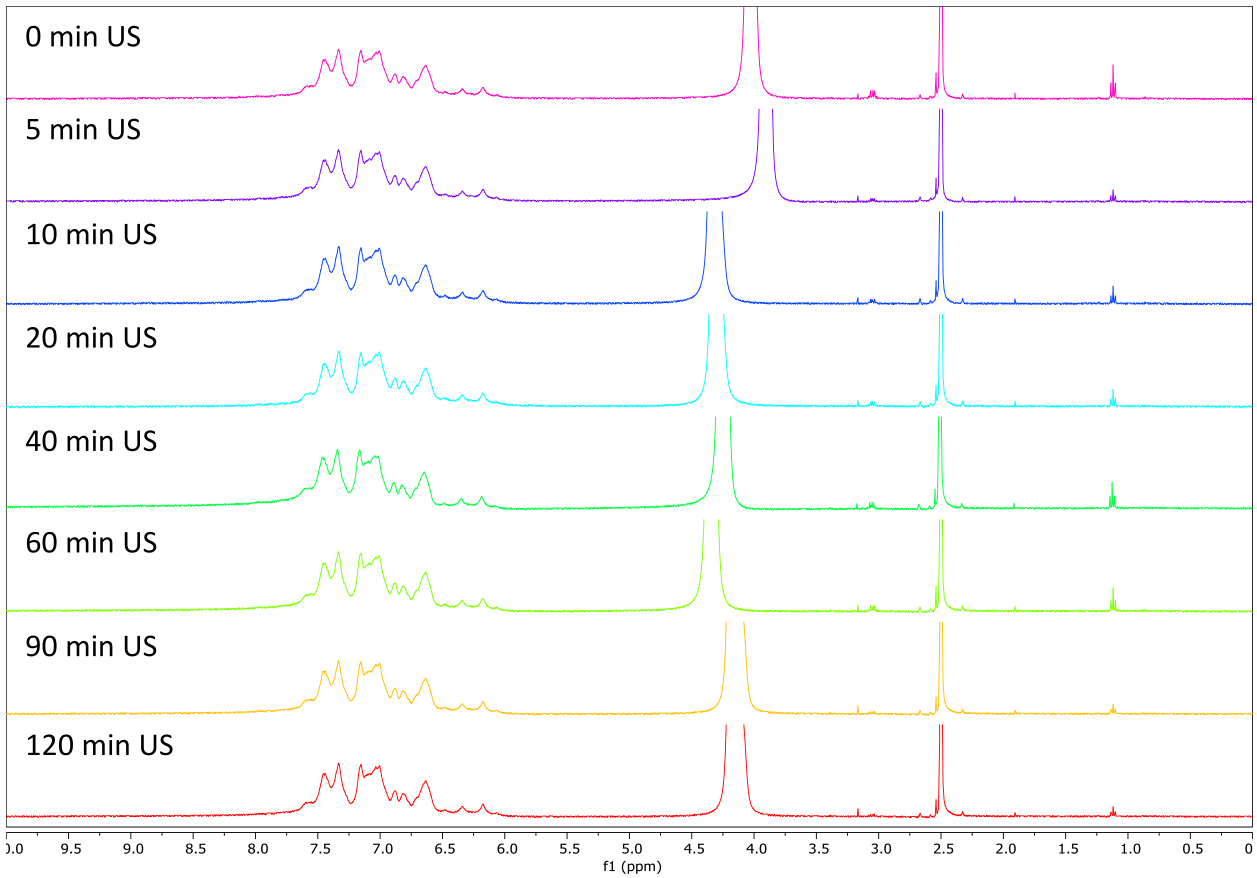

Figure S8. ^{1}H NMR spectra of sPPB-H$^+$ (0.30 wt% solutions) following ultrasound treatments from 0 – 120 min.

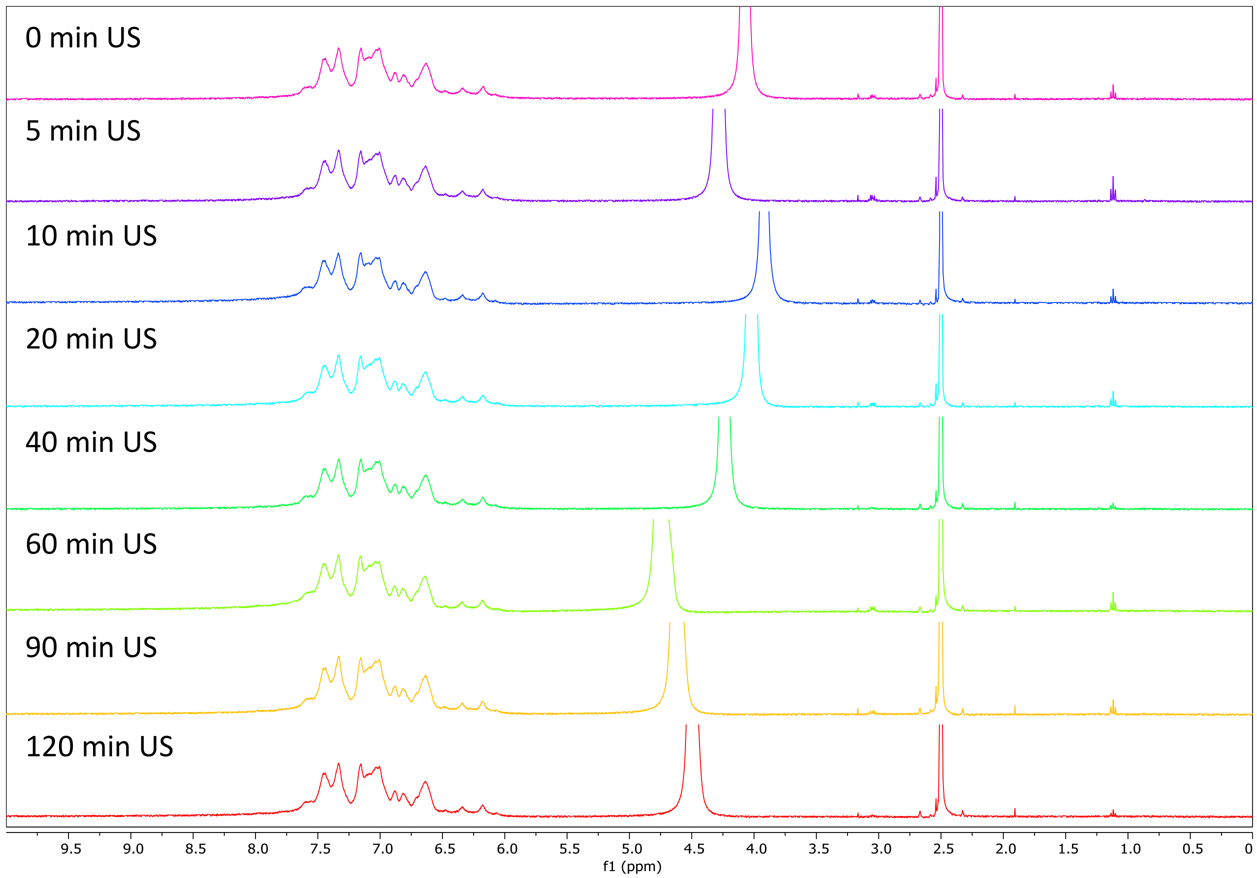

Figure S9. ^1H NMR spectra of sPPB-H$^+$ (1.00 wt% solutions) following ultrasound treatments from 0 – 120 min.

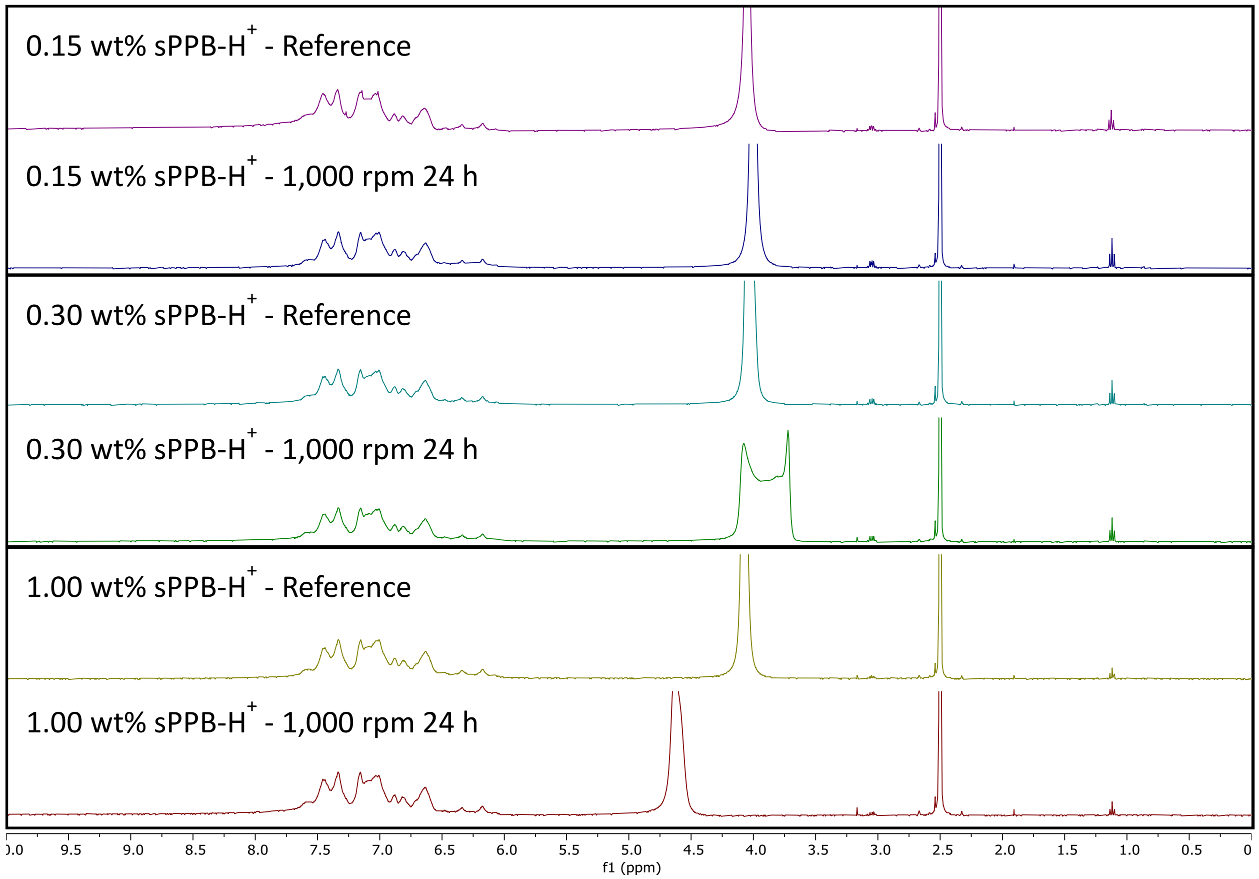

Figure S10. ^{1}H NMR spectra of sPPB-H^{+} (all solutions) following rapid stirring (1,000 rpm, 24 h).

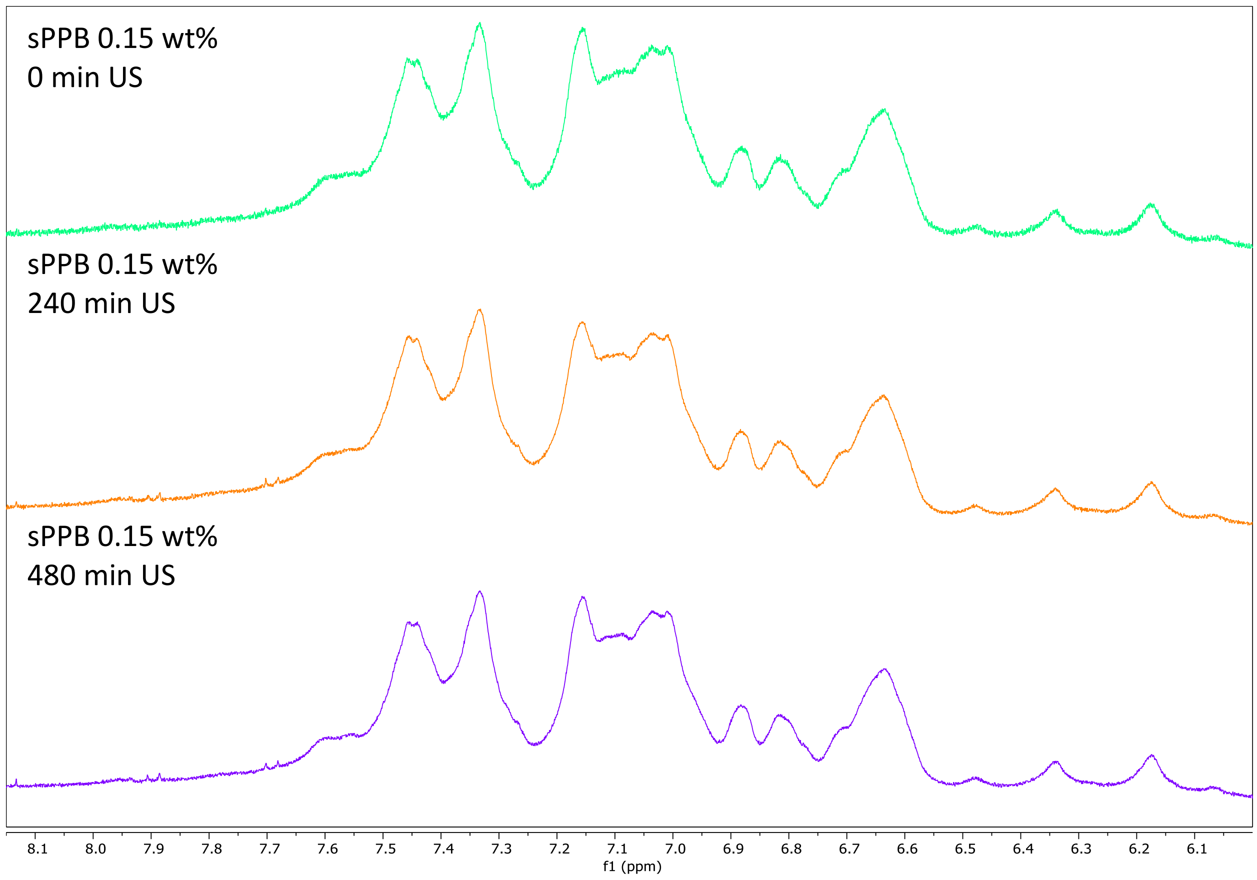

Figure S11. Aromatic regions of 0.15 wt% sPPB-H^+ solution ^1H NMR spectra following 0, 240, and 480 min ultrasonication.

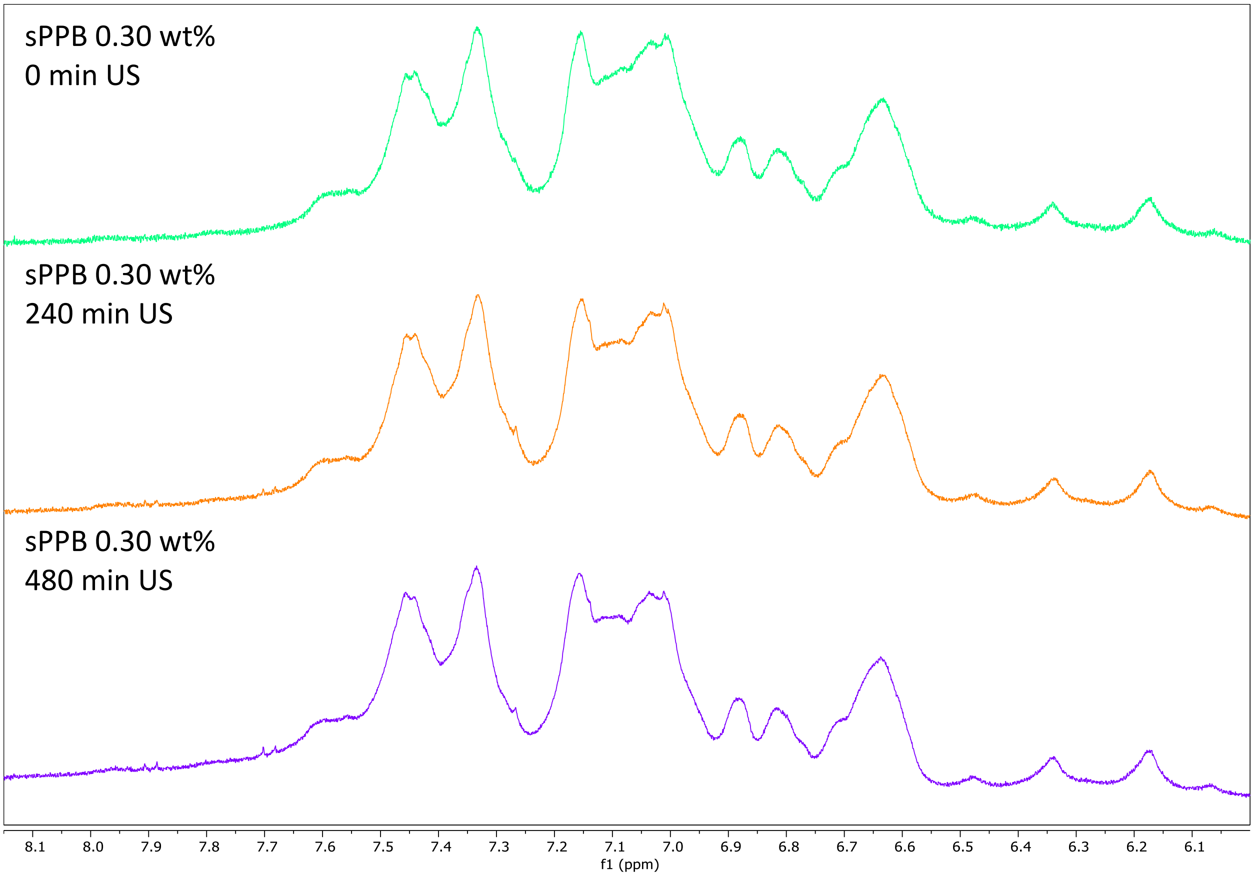

Figure S12. Aromatic regions of 0.30 wt% sPPB-H^+ solution ^{1}H NMR spectra following 0, 240, and 480 min ultrasonication.

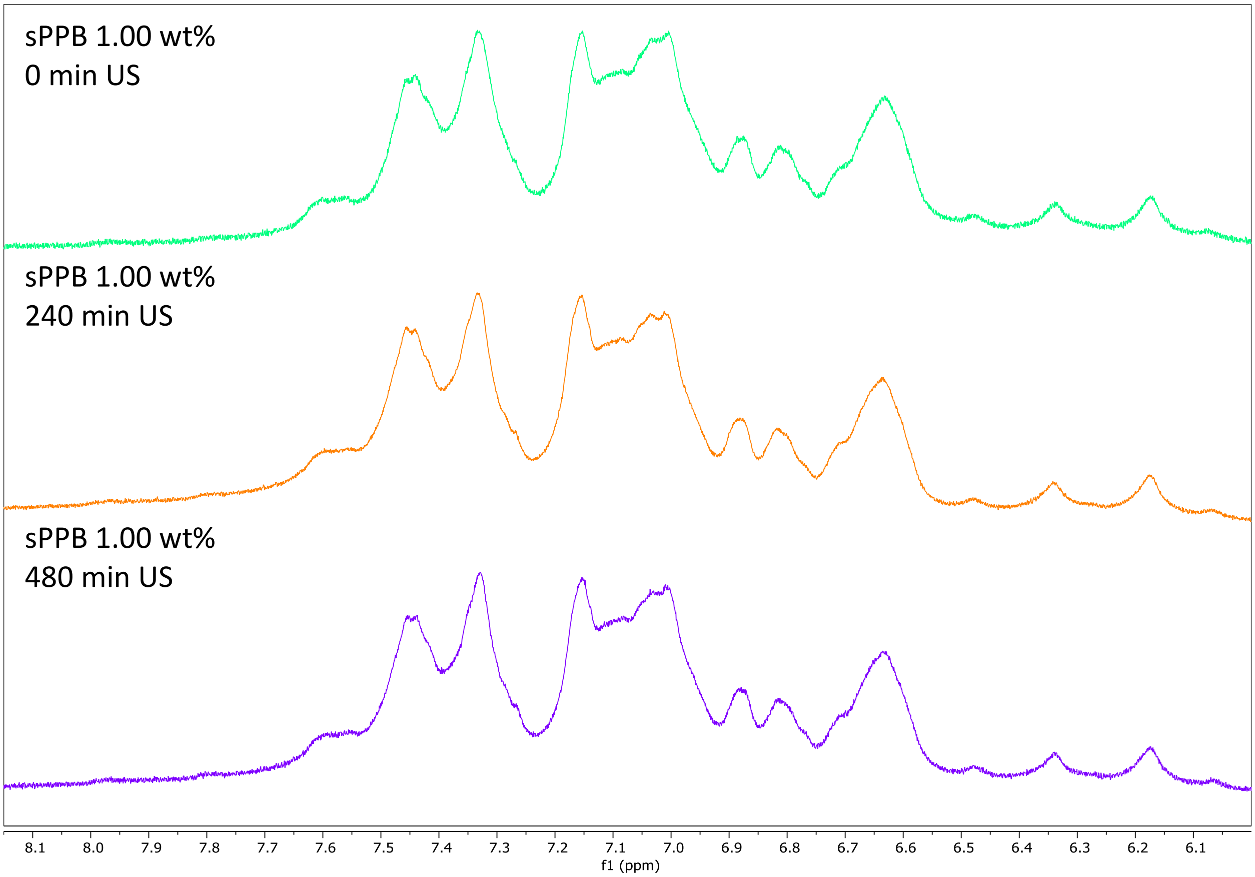

Figure S13. Aromatic regions of 1.00 wt% sPPB-H^+ solution ^{1}H NMR spectra following 0, 240, and 480 min ultrasonication.

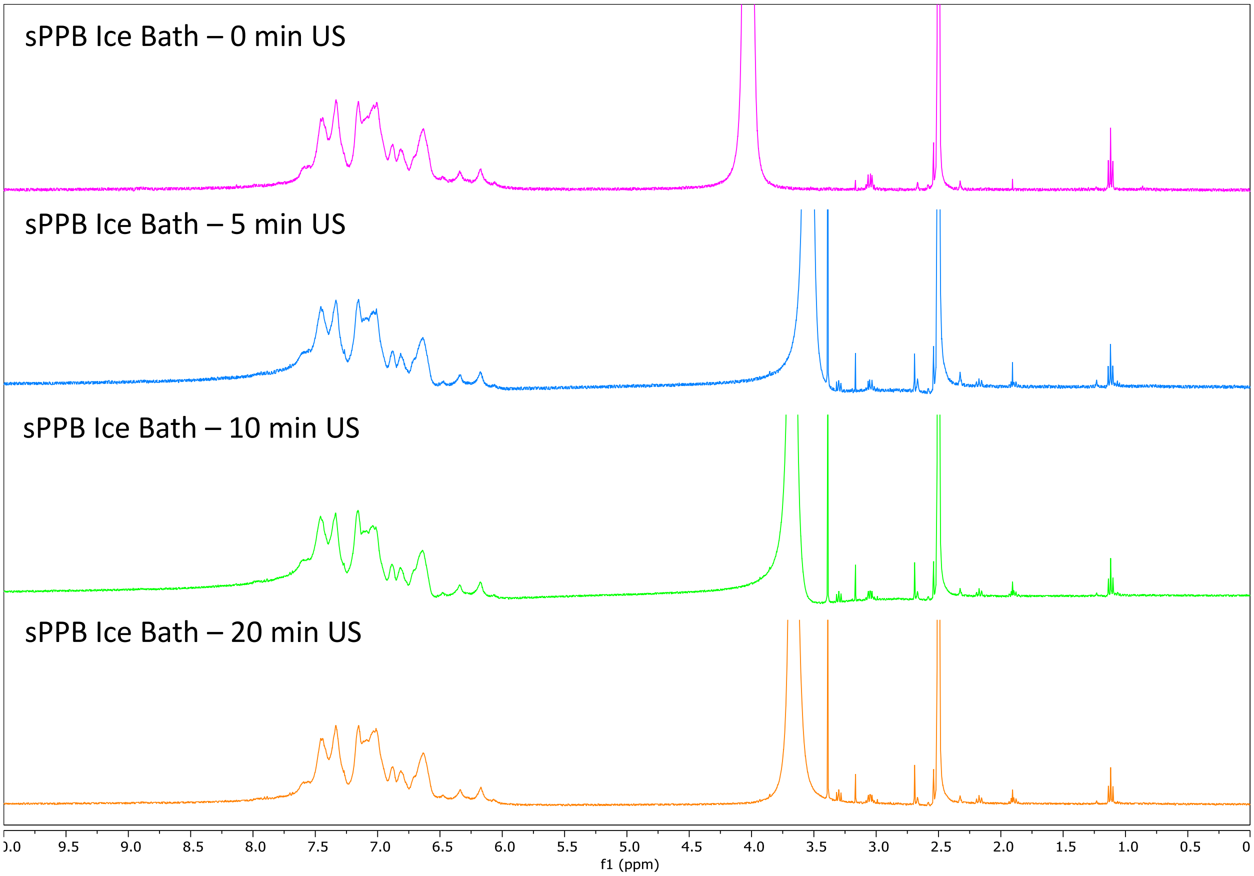

Figure S14. ^{1}H NMR spectra of sPPB-H^{+} (0.30 wt% solutions) following ultrasonication for 0 – 20 min in an ice bath. The additional upfield proton signals between 3.39 and 2.69 ppm found in 5, 10, and 20 min samples are due to impurities from NMR sample vial caps, because PTFE caps were unavailable due to COVID-19-associated supply chain shortages.

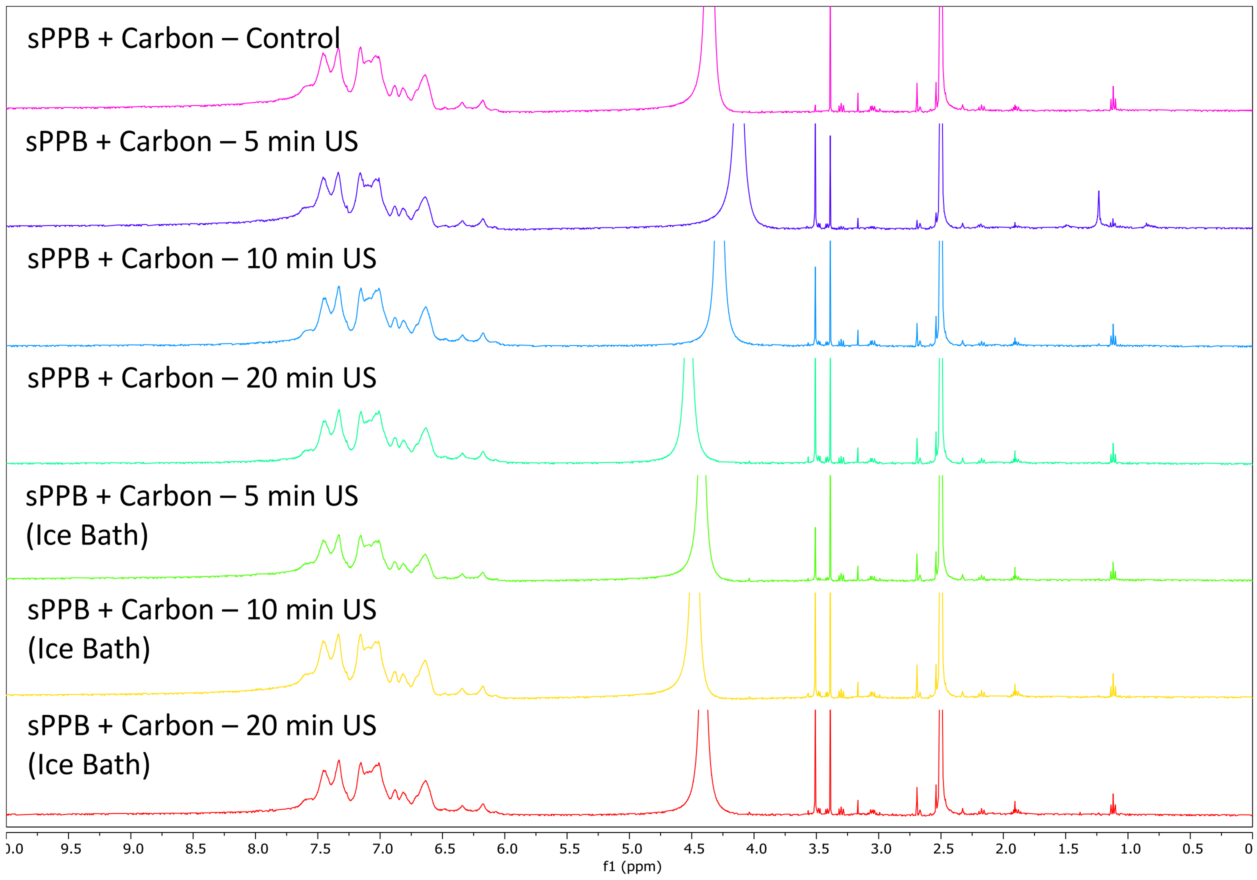

Figure S15. ^{1}H NMR spectra of sPPB-H^{+} (0.30 wt% solutions) following ultrasonication for 0 – 20 min with added carbon black under ambient conditions and in an ice bath. The additional upfield proton signals between 3.50 and 2.69 ppm found in all samples are due to impurities from NMR sample vial caps, because PTFE caps were unavailable due to COVID-19-associated supply chain shortages.

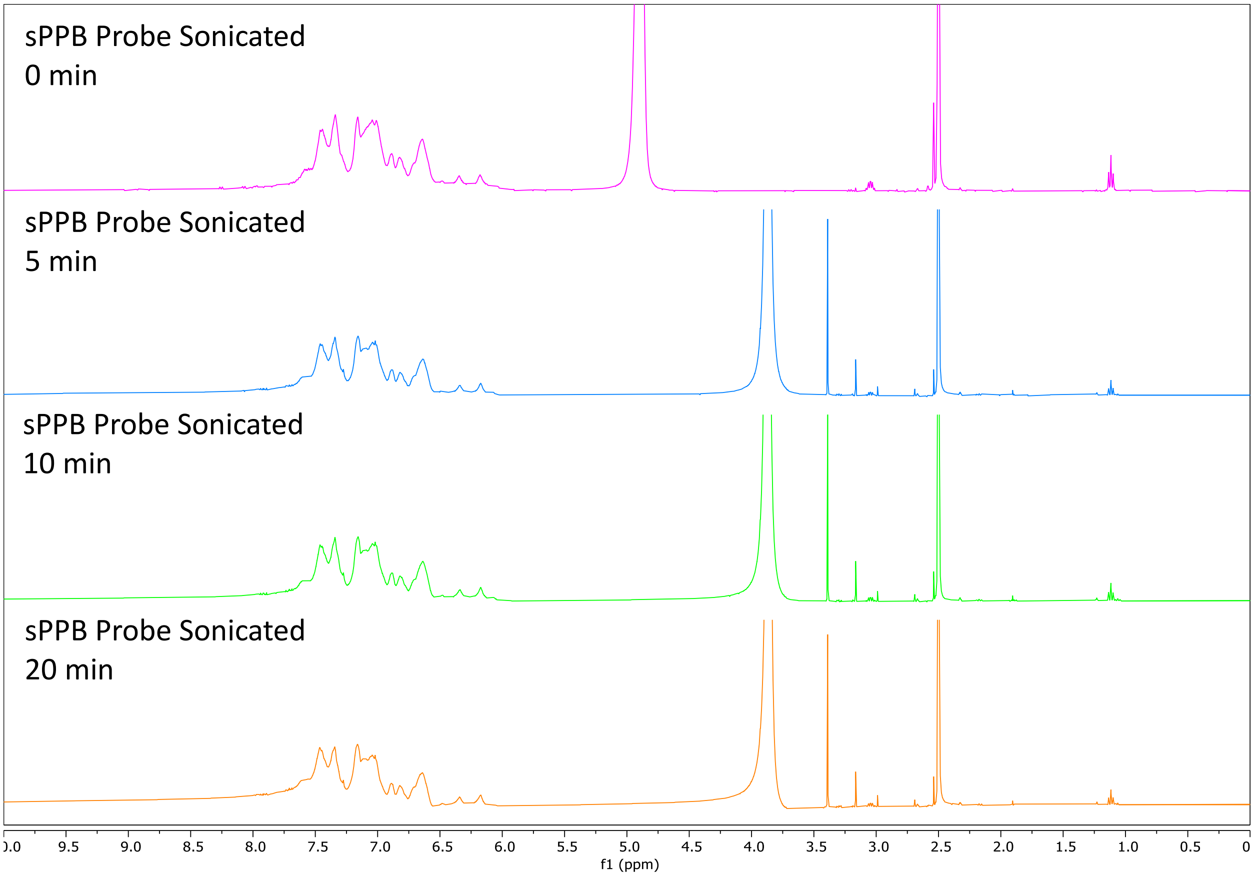

Figure S16. ^{1}H NMR spectra of sPPB-H$^+$ (0.30 wt% solutions) following probe ultrasonication for 0 – 20 min. The additional upfield proton signals between 3.39 and 2.69 ppm found in 5, 10, and 20 min samples are due to impurities from NMR sample vial caps, because PTFE caps were unavailable due to COVID-19-associated supply chain shortages.

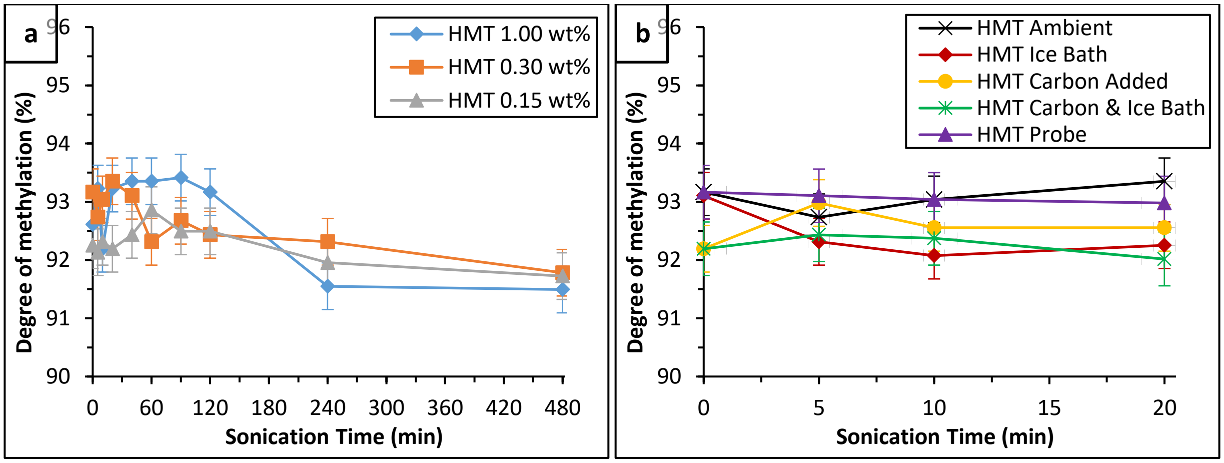

Figure S17. Calculated degree of methylation for HMT-PMBI polymer solutions subject to (a) ultrasonication for 0 – 480 min (0.15, 0.30, and 1.00 wt% in 3:1 MeOH/H_2O), and (b) 0.30 wt% in 3:1 MeOH/H_2O solutions following sample ultrasonication for 0 – 20 min at ambient temperature, in an ice bath, with solutions containing added carbon black, in an ice bath with solutions containing added carbon black, and using a probe sonicator (26 kHz, 10.6 W). Error bars = standard deviation (n = 3).

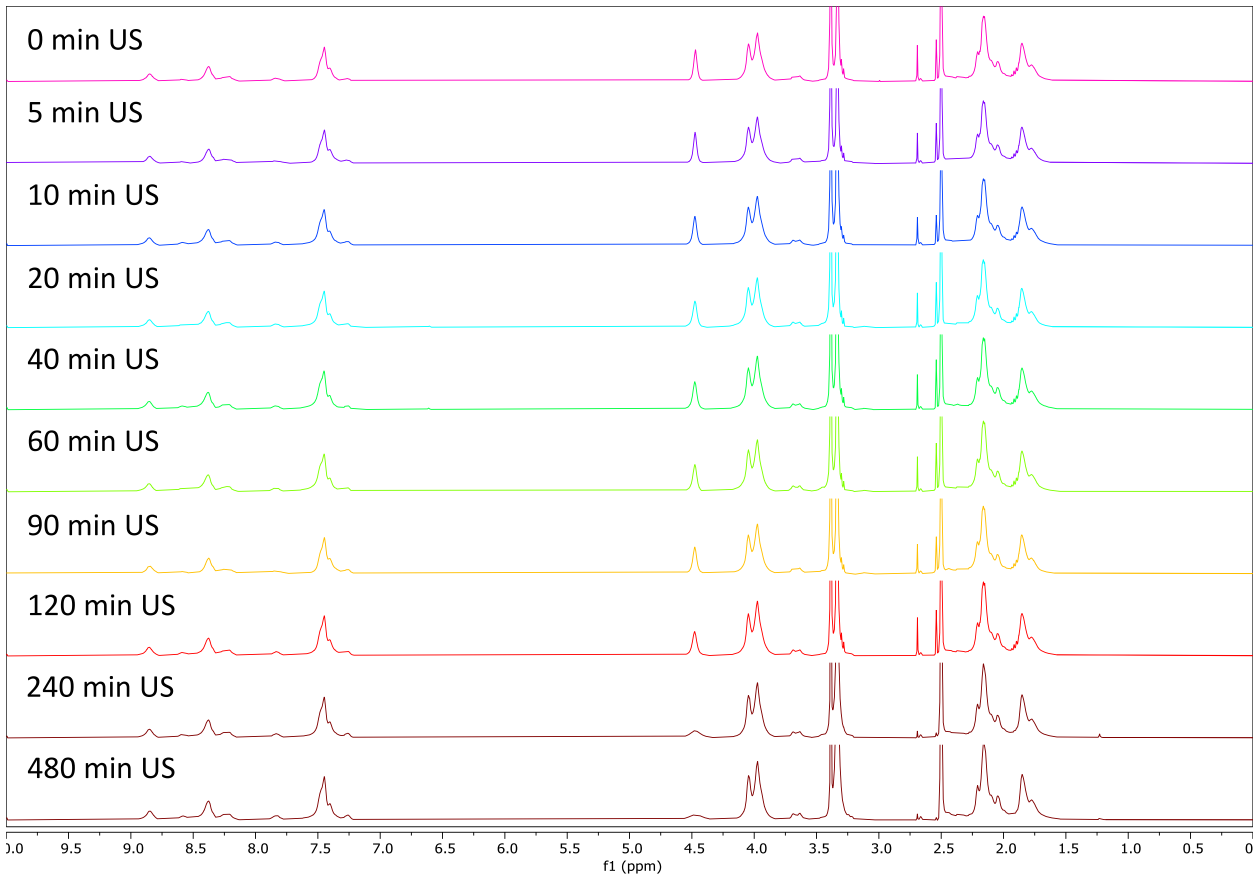

Figure S18. ^{1}H NMR spectra of HMT-PMBI (0.15 wt% solutions) following ultrasound treatments from 0 – 480 min.

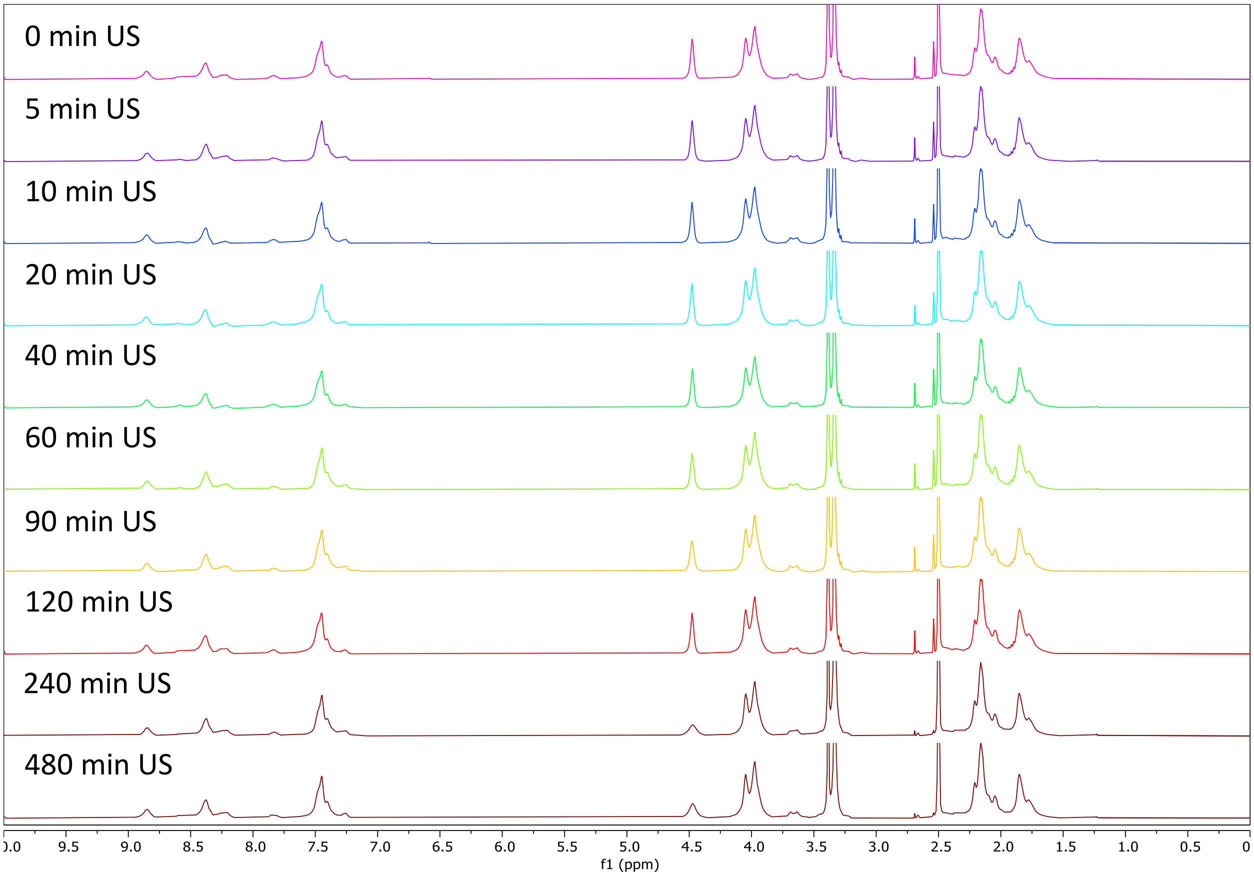

Figure S19. ^{1}H NMR spectra of HMT-PMBI (0.30 wt% solutions) following ultrasound treatments from 0 – 480 min.

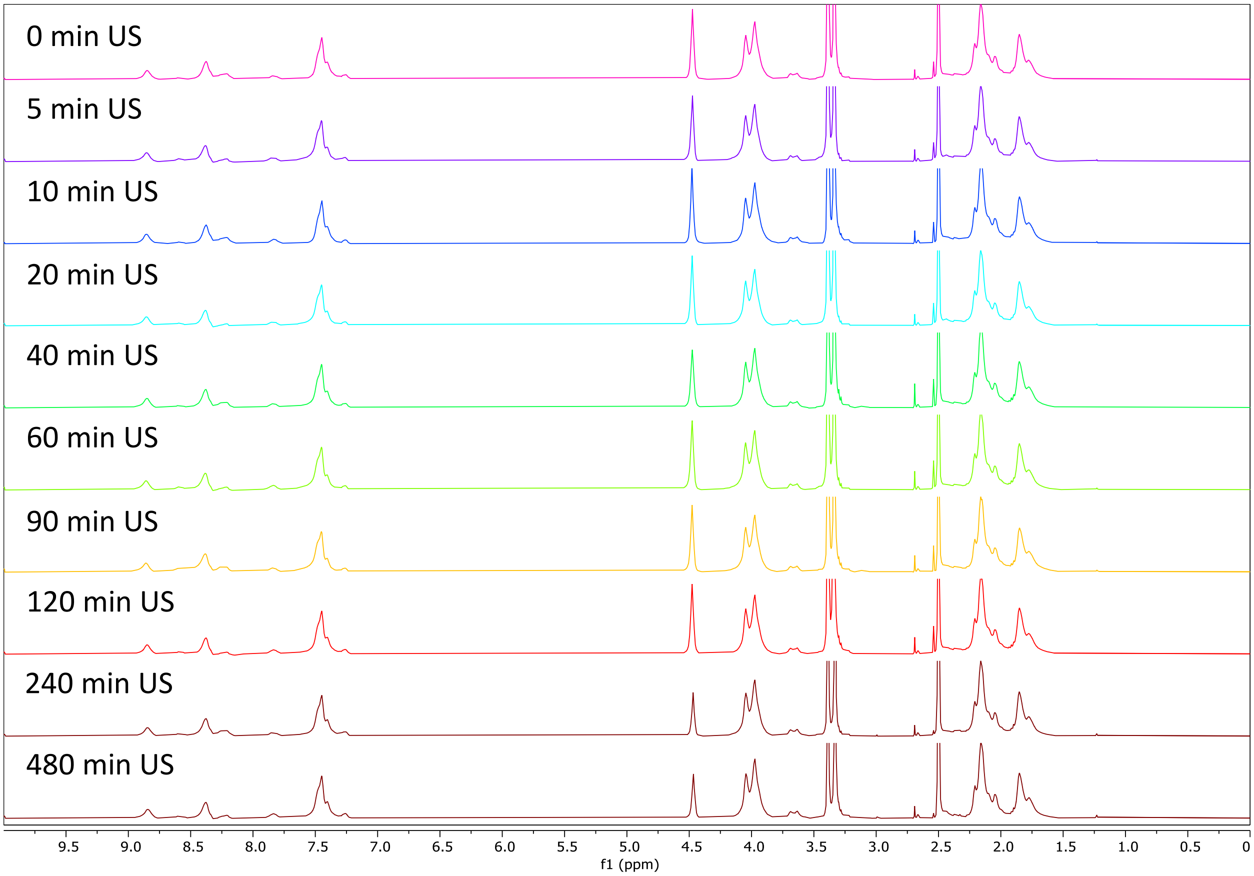

Figure S20. ^{1}H NMR spectra of HMT-PMBI (1.00 wt% solutions) following ultrasound treatments from 0 – 480 min.

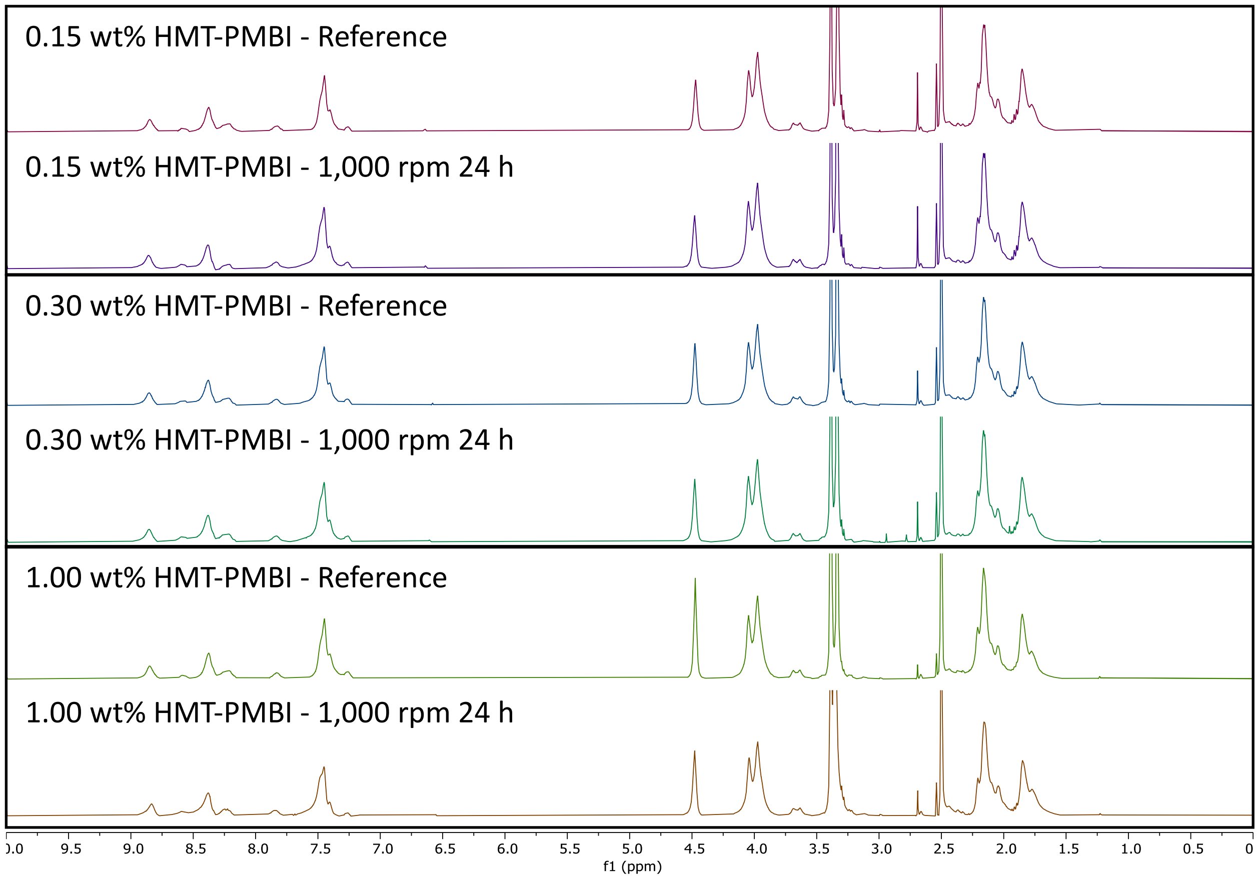

Figure S21. ^{1}H NMR spectra of HMT-PMBI (all solutions) following rapid stirring (1,000 rpm, 24 h).

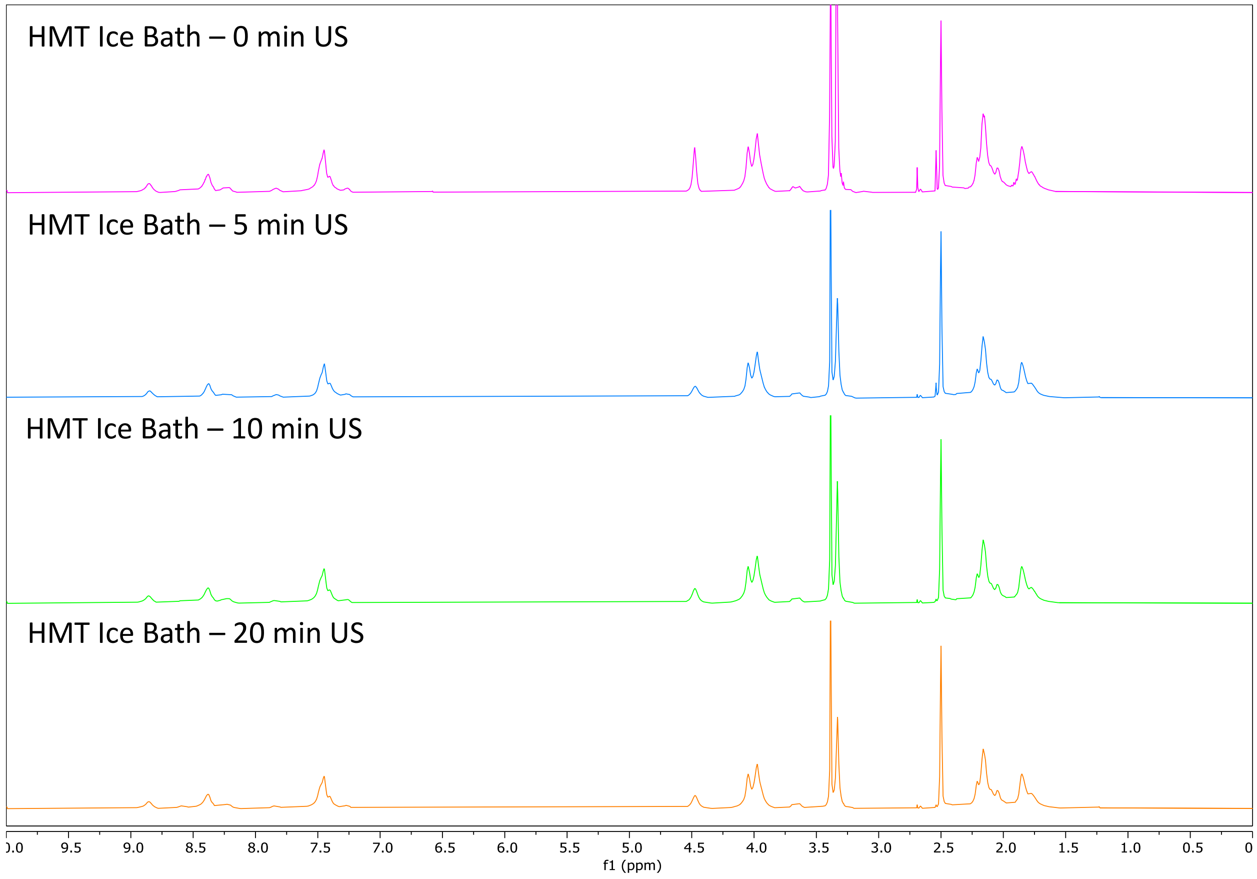

Figure S22. ^{1}H NMR spectra of HMT-PMBI (0.30 wt% solutions) following ultrasonication for 0 – 20 min in an ice bath. The additional upfield proton signals between 3.39 and 2.69 ppm found in 5, 10, and 20 min samples are due to impurities from NMR sample vial caps, because PTFE caps were unavailable due to COVID-19-associated supply chain shortages.

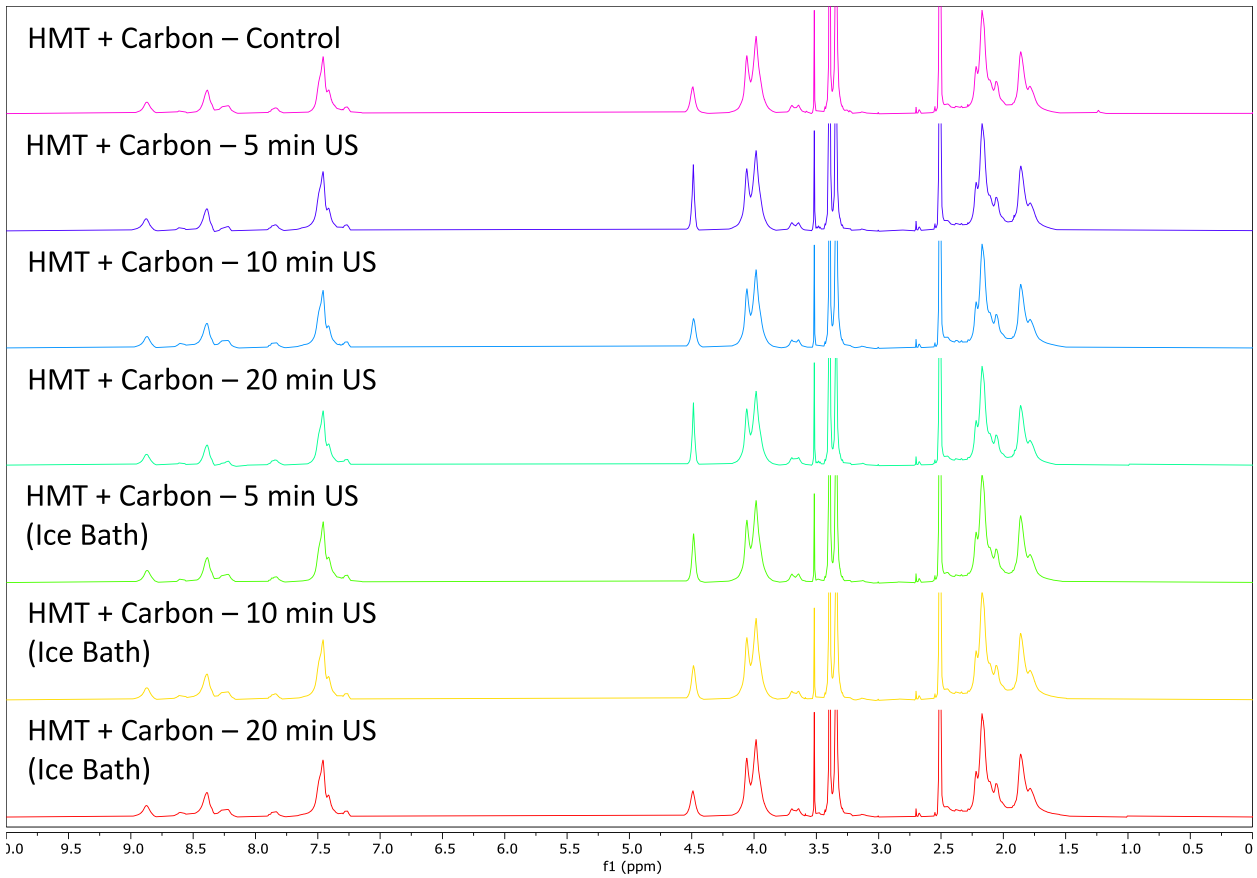

Figure S23. ^{1}H NMR spectra of HMT-PMBI (0.30 wt% solutions) following ultrasonication for 0 – 20 min with added carbon black under ambient conditions and in an ice bath. The additional upfield proton signals between 3.50 and 2.69 ppm found in all samples are due to impurities from NMR sample vial caps, because PTFE caps were unavailable due to COVID-19-associated supply chain shortages.

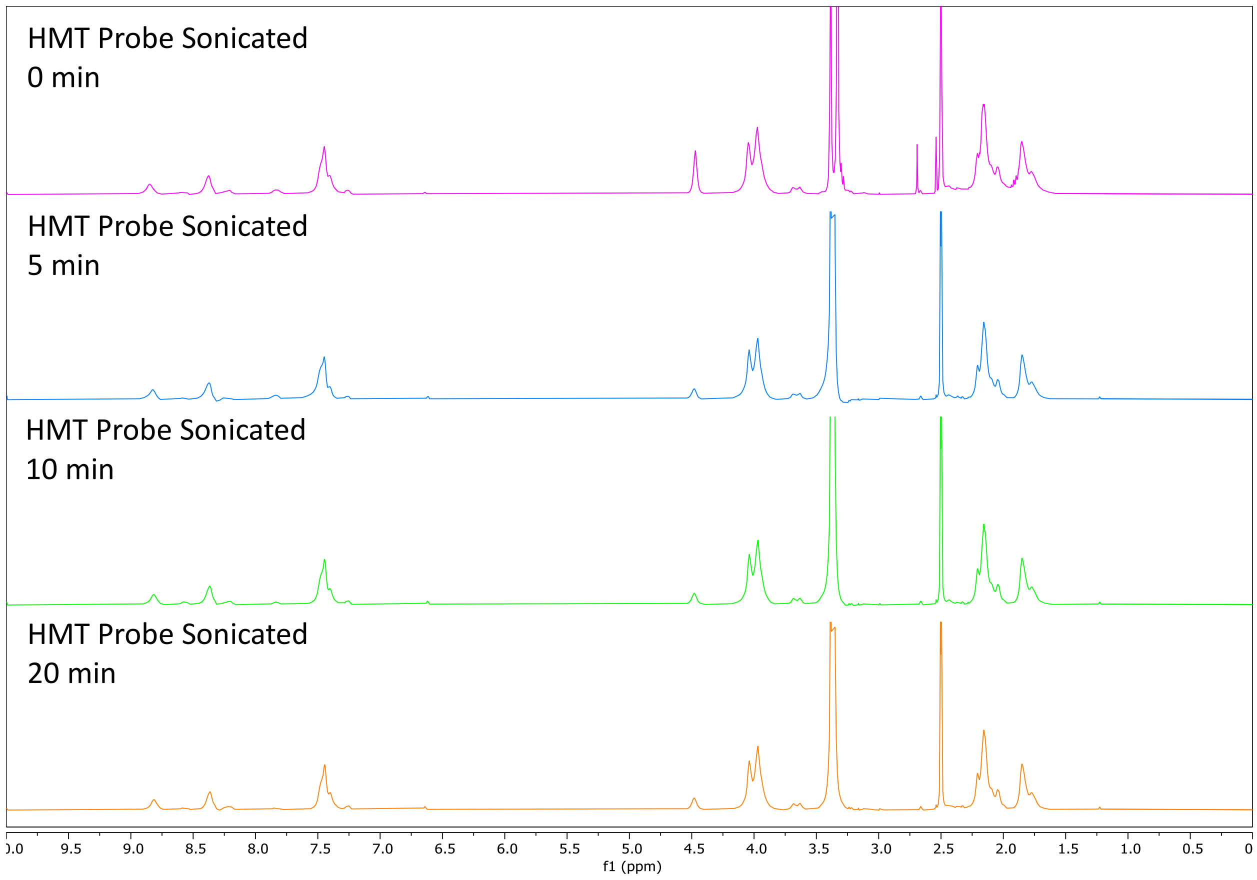

Figure S24. ^1H NMR spectra of HMT-PMBI (0.30 wt% solutions) following probe ultrasonication for 0 – 20 min.

In-situ Characterization Data

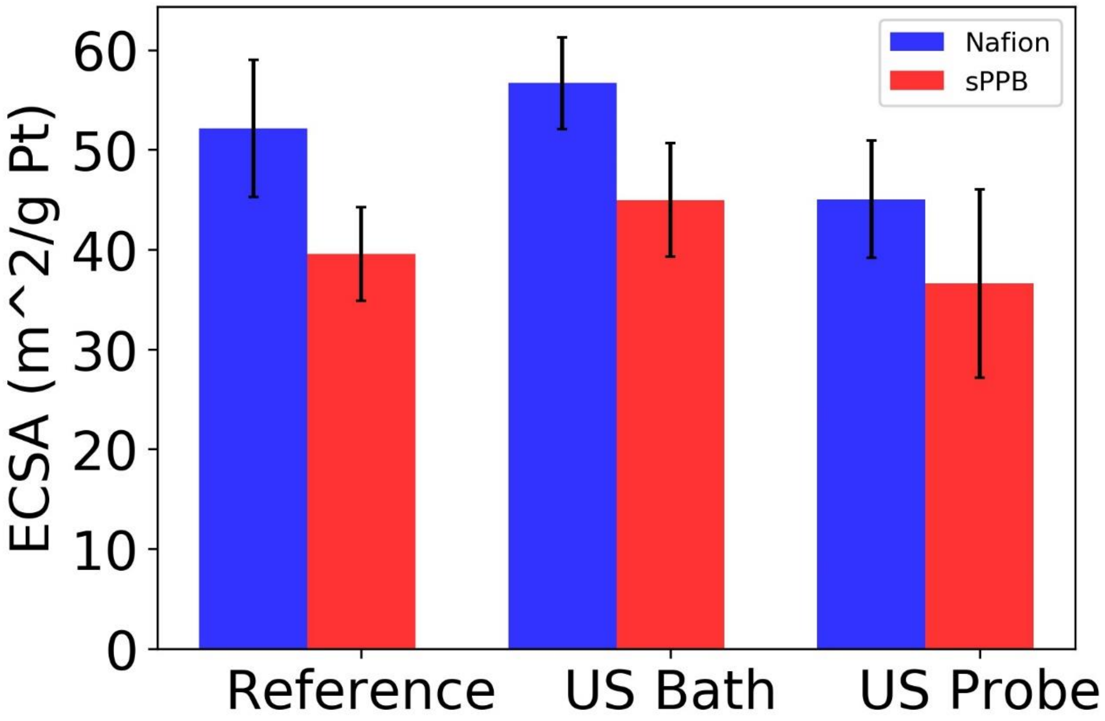

Figure S25. ECSA data extracted from the CV analysis of MEAs containing Nafion® (blue) or sPPB-H^+ (red) ionomer in the catalyst layer. Polymer solutions used in MEA preparation were pre-treated for 20 min with either an ultrasonication bath (US Bath) or probe (US Probe), or left untreated (reference). CV scans were performed at 80 ^oC, H_2 anode and N_2 cathode, 100% RH, 1 atm pressure. Error bars represent the standard deviation of n = 3 independently fabricated and assessed MEAs.

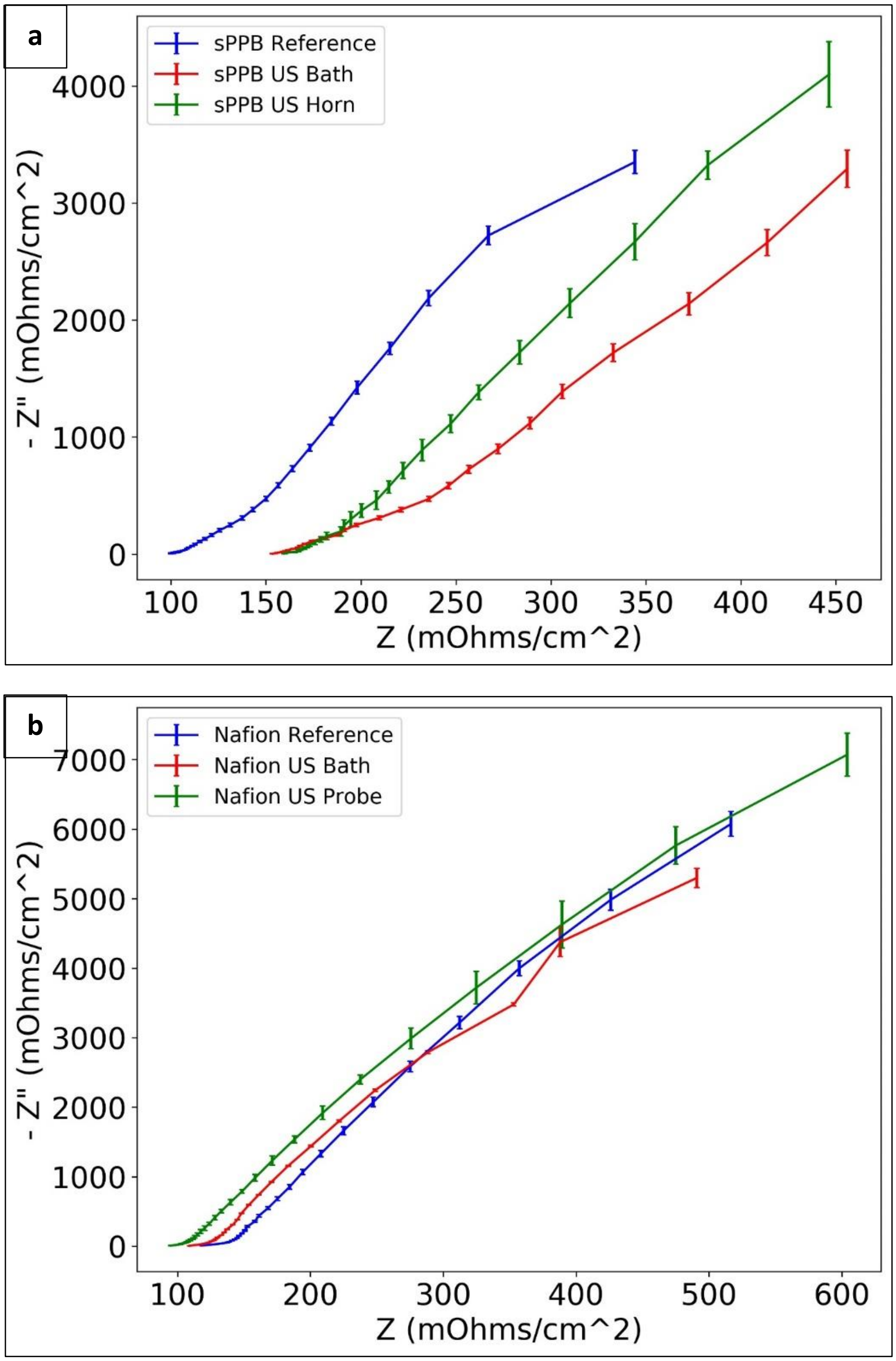

Figure S26. Nyquist plots illustrating the high-frequency intercept and low-frequency intercept by a linear fit of low-frequency data, used to determine catalyst layer ionic resistance of MEAs containing (a) sPPB-H^+, or (b) Nafion® ionomer in the catalyst layer. Polymer solutions used in MEA preparation were pre-treated for 20 min with either an ultrasonication bath (US Bath) or probe (US Probe), or left untreated (reference). Characterization conditions were 80°C, H_2 anode and N_2 cathode gases, 100% RH, 1 atm pressure. Error bars represent the standard deviation of n = 3 independently fabricated and assessed MEAs.

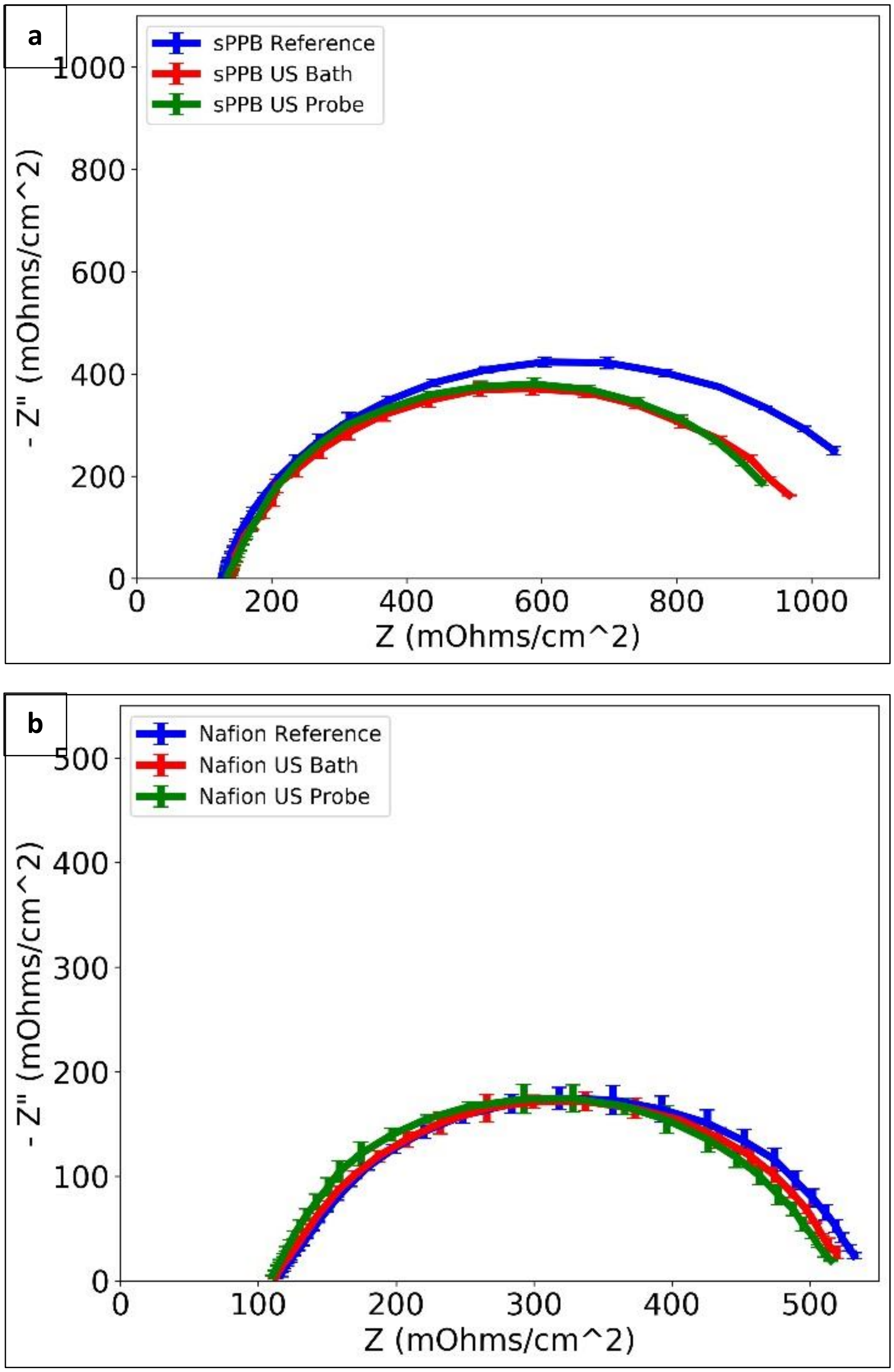

Figure S27. Nyquist plots of MEAs containing (a) sPPB-H^+, or (b) Nafion® ionomer in the catalyst layer. Polymer solutions used in MEA preparation were pre-treated for 20 min with either an ultrasonication bath (US Bath) or probe (US Probe), or left untreated (reference). EIS spectra recorded were at 0.8 V, 80°C, H_2 anode and O_2 cathode, 100% RH, 1 atm pressure, and data was used to calculate charge transfer resistance, R_{ct}. Error bars represent the standard deviation of n = 3 independently fabricated and assessed MEAs.

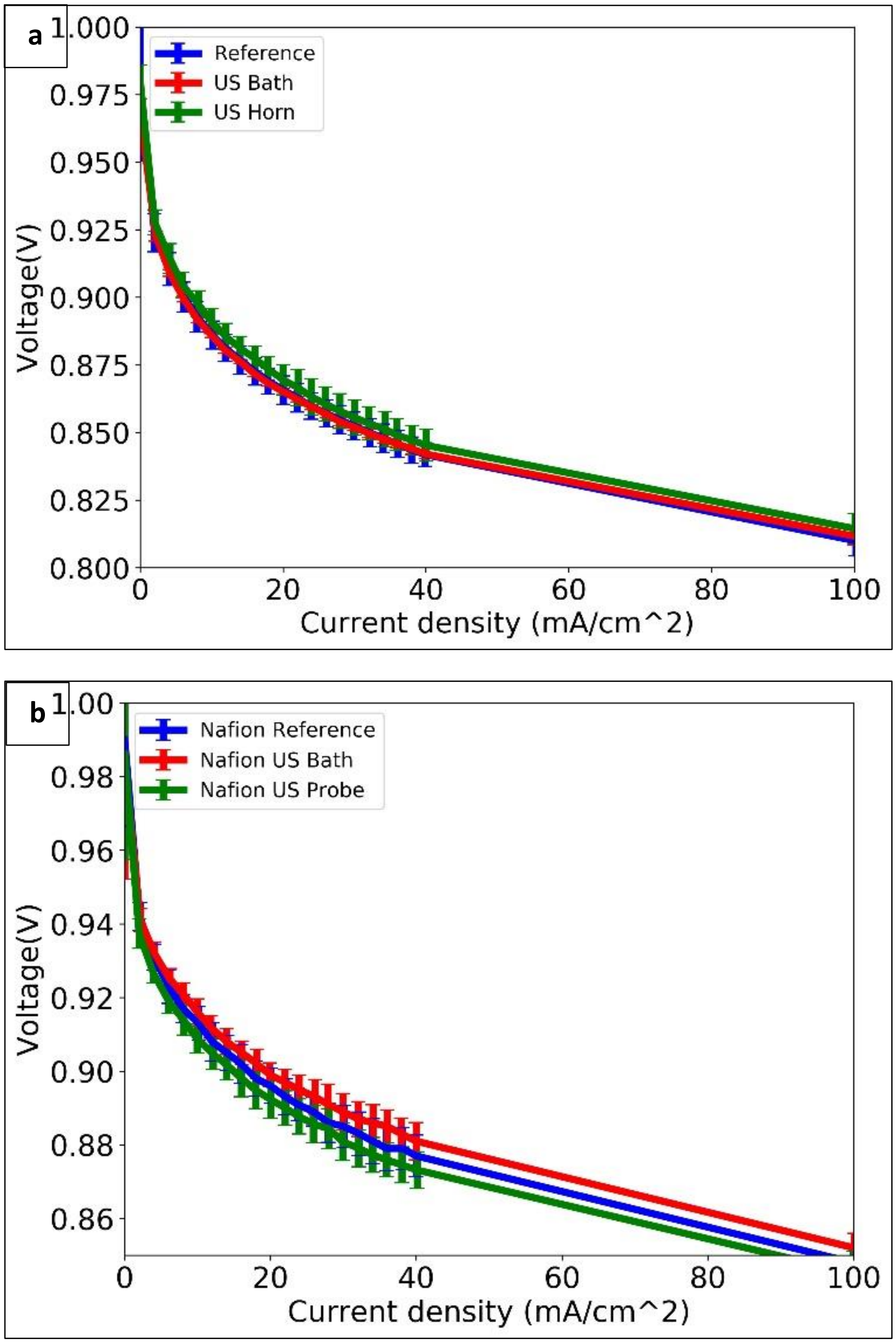

Figure S28. Polarization curves for the low current density region (activation region, < 100 mA·cm^{-2}) of MEAs containing (a) sPPB-H^{+}, or (b) Nafion® ionomer in the catalyst layer. Polymer solutions used in MEA preparation were pre-treated for 20 min with either an ultrasonication bath (US Bath) or probe (US Probe), or left untreated (reference). Characterization conditions were 80°C, H_2 anode and N_2 cathode gases, 100% RH, 1 atm pressure. Error bars represent the standard deviation of n = 3 independently fabricated and assessed MEAs.

T-Test Statistically Significant Analyses

Table S1. Nafion polarization curves *T*-test – Reference versus US Bath. *T*-tests were performed for each data point to 95% confidence limits, where $p < 0.05$ indicates statistically different values.

	Power Density Curve				Voltage Curve			
**Current Density**	**Degrees of Freedom**	***t*-value**	***p*-value**	**Statistically Different?**	**Degrees of Freedom**	***t*-value**	***p*-value**	**Statistically Different?**
2.0E+00	2.2	0.6	0.63	No	3.9	0.4	0.75	No
4.0E+00	2.4	1.7	0.24	No	3.9	0.4	0.73	No
6.0E+00	2.8	3.3	0.08	No	3.3	0.6	0.57	No
8.0E+00	3.7	0.7	0.54	No	3.8	1.4	0.25	No
1.0E+01	2.9	0.0	1.00	No	3.8	0.9	0.44	No
1.2E+01	2.2	0.0	1.00	No	3.2	1.2	0.33	No
1.4E+01	3.1	1.3	0.29	No	3.7	0.9	0.45	No
1.6E+01	3.6	2.1	0.12	No	3.3	0.8	0.46	No
1.8E+01	3.0	2.6	0.12	No	3.9	1.1	0.35	No
2.0E+01	3.1	1.4	0.27	No	3.5	0.9	0.43	No
2.2E+01	3.7	0.0	1.00	No	3.6	1.2	0.33	No
2.4E+01	2.8	1.5	0.28	No	3.9	1.2	0.33	No
2.6E+01	2.6	2.8	0.11	No	3.9	1.1	0.34	No
2.8E+01	3.9	0.0	1.00	No	4.0	1.1	0.34	No
3.0E+01	3.4	1.8	0.17	No	3.9	0.9	0.43	No
3.2E+01	3.5	1.5	0.22	No	3.8	0.9	0.43	No
3.4E+01	4.0	1.6	0.21	No	3.8	1.1	0.35	No
3.6E+01	3.4	1.7	0.18	No	3.7	1.4	0.26	No
3.8E+01	3.3	1.2	0.31	No	3.7	1.0	0.40	No
4.0E+01	4.0	1.6	0.21	No	3.9	0.9	0.43	No
1.0E+02	3.8	2.1	0.13	No	3.9	1.1	0.35	No
2.0E+02	4.0	2.8	0.07	No	3.9	1.9	0.16	No
3.0E+02	4.0	3.6	0.04	**Yes**	3.9	2.8	0.07	No
4.0E+02	3.6	3.4	0.04	**Yes**	3.8	2.6	0.08	No
6.0E+02	3.8	4.2	0.02	**Yes**	3.7	3.7	0.03	**Yes**
8.0E+02	3.5	4.2	0.02	**Yes**	2.1	4.4	0.05	**Yes**
1.0E+03	3.3	3.5	0.04	**Yes**	2.1	3.4	0.08	No
1.2E+03	4.0	3.6	0.04	**Yes**	2.4	4.0	0.06	No
1.4E+03	3.9	4.0	0.03	**Yes**	3.5	4.4	0.02	**Yes**
1.6E+03	3.9	3.6	0.04	**Yes**	3.5	4.0	0.03	**Yes**
1.8E+03	3.4	3.9	0.03	**Yes**	4.0	4.4	0.01	**Yes**
2.0E+03	4.0	3.9	0.03	**Yes**	3.6	3.8	0.03	**Yes**
2.2E+03	4.0	3.2	0.05	No	3.9	3.4	0.04	**Yes**
2.4E+03	3.6	2.5	0.09	No	4.0	2.7	0.07	No
2.6E+03	4.0	2.7	0.08	No	3.9	3.0	0.06	No
2.8E+03	3.8	2.2	0.12	No	4.0	2.2	0.12	No
3.0E+03	3.3	2.4	0.10	No	3.8	2.3	0.10	No

Table S2. Nafion polarization curves *T*-test – Reference versus US Probe. *T*-tests were performed for each data point to 95% confidence limits, where $p < 0.05$ indicates statistically different values.

	Power Density Curve				Voltage Curve			
**Current Density**	**Degrees of Freedom**	***t*-value**	***p*-value**	**Statistically Different?**	**Degrees of Freedom**	***t*-value**	***p*-value**	**Statistically Different?**
2.0E+00	2.6	0.3	0.79	No	4.0	1.5	0.23	No
4.0E+00	2.4	0.2	0.85	No	3.7	1.8	0.18	No
6.0E+00	2.6	0.9	0.48	No	3.7	1.1	0.34	No
8.0E+00	3.5	0.6	0.60	No	4.0	1.0	0.39	No
1.0E+01	2.7	1.8	0.22	No	3.9	1.2	0.33	No
1.2E+01	2.3	2.0	0.18	No	3.8	0.9	0.45	No
1.4E+01	3.8	1.7	0.19	No	3.9	0.9	0.44	No
1.6E+01	3.9	0.0	1.00	No	4.0	0.9	0.42	No
1.8E+01	2.6	0.0	1.00	No	4.0	0.8	0.49	No
2.0E+01	3.4	2.4	0.10	No	4.0	0.9	0.43	No
2.2E+01	2.9	0.9	0.45	No	4.0	0.8	0.50	No
2.4E+01	3.2	0.0	1.00	No	4.0	0.9	0.45	No
2.6E+01	3.6	0.0	1.00	No	3.7	0.9	0.43	No
2.8E+01	3.9	0.0	1.00	No	4.0	0.4	0.74	No
3.0E+01	3.0	0.0	1.00	No	4.0	0.9	0.42	No
3.2E+01	3.6	0.8	0.49	No	3.9	0.9	0.44	No
3.4E+01	3.9	0.9	0.45	No	3.8	0.8	0.46	No
3.6E+01	4.0	0.5	0.67	No	3.8	0.7	0.53	No
3.8E+01	3.3	0.6	0.59	No	3.9	1.0	0.38	No
4.0E+01	3.3	0.0	1.00	No	3.9	0.9	0.44	No
1.0E+02	3.9	0.0	1.00	No	3.8	0.7	0.56	No
2.0E+02	4.0	0.0	1.00	No	4.0	0.6	0.58	No
3.0E+02	3.7	0.0	1.00	No	3.8	0.6	0.59	No
4.0E+02	3.3	0.0	1.00	No	3.6	0.7	0.54	No
6.0E+02	3.4	0.0	1.00	No	3.2	0.5	0.68	No
8.0E+02	3.0	0.0	1.00	No	2.1	0.3	0.78	No
1.0E+03	3.3	0.6	0.61	No	2.1	1.2	0.36	No
1.2E+03	3.8	0.2	0.86	No	2.3	0.9	0.46	No
1.4E+03	3.7	0.0	1.00	No	2.7	0.4	0.73	No
1.6E+03	3.8	0.0	1.00	No	2.9	0.1	0.93	No
1.8E+03	3.9	0.8	0.50	No	3.1	0.2	0.84	No
2.0E+03	3.9	0.0	1.00	No	3.1	0.2	0.85	No
2.2E+03	3.1	0.5	0.64	No	2.7	0.8	0.53	No
2.4E+03	3.8	0.0	1.00	No	3.0	0.5	0.67	No
2.6E+03	4.0	0.0	1.00	No	3.9	0.0	0.97	No
2.8E+03	3.9	0.3	0.81	No	3.6	0.0	0.98	No
3.0E+03	3.9	0.2	0.85	No	3.9	0.5	0.67	No

Table S3. Nafion polarization curves *T*-test – US Bath versus US Probe. *T*-tests were performed for each data point to 95% confidence limits, where $p < 0.05$ indicates statistically different values.

	Power Density Curve				Voltage Curve			
**Current Density**	**Degrees of Freedom**	***t*-value**	***p*-value**	**Statistically Different?**	**Degrees of Freedom**	***t*-value**	***p*-value**	**Statistically Different?**
2.0E+00	3.2	0.3	0.76	No	3.8	1.3	0.30	No
4.0E+00	4.0	1.4	0.25	No	3.9	2.3	0.10	No
6.0E+00	4.0	1.7	0.19	No	3.8	2.2	0.11	No
8.0E+00	2.9	1.2	0.34	No	3.7	2.5	0.09	No
1.0E+01	3.9	1.5	0.24	No	4.0	2.3	0.11	No
1.2E+01	3.9	1.4	0.26	No	3.6	2.4	0.09	No
1.4E+01	3.5	2.4	0.10	No	3.9	2.0	0.14	No
1.6E+01	3.9	2.4	0.09	No	3.4	2.0	0.14	No
1.8E+01	3.8	1.8	0.17	No	3.8	1.9	0.15	No
2.0E+01	2.5	2.9	0.10	No	3.4	1.9	0.15	No
2.2E+01	3.5	0.9	0.45	No	3.7	2.1	0.13	No
2.4E+01	3.8	2.2	0.11	No	3.9	2.0	0.13	No
2.6E+01	2.3	2.1	0.18	No	4.0	1.9	0.16	No
2.8E+01	4.0	0.0	1.00	No	4.0	1.5	0.23	No
3.0E+01	3.9	1.4	0.26	No	4.0	2.0	0.14	No
3.2E+01	4.0	2.0	0.14	No	4.0	2.0	0.14	No
3.4E+01	3.9	2.6	0.08	No	4.0	2.2	0.11	No
3.6E+01	3.2	2.2	0.12	No	4.0	2.4	0.09	No
3.8E+01	4.0	1.5	0.23	No	3.9	2.3	0.11	No
4.0E+01	3.3	1.9	0.15	No	4.0	1.9	0.15	No
1.0E+02	3.5	1.9	0.15	No	3.5	1.7	0.20	No
2.0E+02	3.9	2.7	0.07	No	3.9	2.6	0.08	No
3.0E+02	3.6	3.0	0.06	No	3.6	3.0	0.06	No
4.0E+02	3.9	2.7	0.07	No	3.9	2.8	0.07	No
6.0E+02	3.8	3.3	0.04	**Yes**	3.8	3.3	0.05	**Yes**
8.0E+02	3.8	3.1	0.05	No	3.8	3.0	0.06	No
1.0E+03	4.0	3.4	0.04	**Yes**	4.0	3.3	0.05	**Yes**
1.2E+03	3.9	3.4	0.04	**Yes**	3.9	3.5	0.04	**Yes**
1.4E+03	3.4	3.3	0.05	**Yes**	3.4	3.2	0.05	**Yes**
1.6E+03	3.6	3.1	0.05	No	3.6	2.9	0.06	No
1.8E+03	3.1	2.7	0.08	No	3.1	2.8	0.07	No
2.0E+03	3.8	3.5	0.04	**Yes**	3.8	3.0	0.06	No
2.2E+03	2.9	2.6	0.12	No	2.9	2.7	0.11	No
2.4E+03	3.0	2.1	0.13	No	3.0	2.3	0.11	No
2.6E+03	4.0	2.8	0.05	No	4.0	2.7	0.07	No
2.8E+03	3.5	1.7	0.18	No	3.5	1.8	0.18	No
3.0E+03	3.5	2.9	0.06	No	3.5	2.6	0.08	No

Table S4. sPPB-H^+ polarization curves *T*-test – Reference versus US Bath. *T*-tests were performed for each data point to 95% confidence limits, where $p < 0.05$ indicates statistically different values.

	**Power Density Curve**				**Voltage Curve**			
**Current Density**	**Degrees of Freedom**	***t*-value**	***p*-value**	**Statistically Different?**	**Degrees of Freedom**	***t*-value**	***p*-value**	**Statistically Different?**
2.0E+00	2.6	1.8	0.21	No	2.3	0.3	0.80	No
4.0E+00	3.9	1.3	0.28	No	2.2	0.4	0.74	No
6.0E+00	2.4	0.3	0.82	No	2.2	0.2	0.84	No
8.0E+00	2.4	0.7	0.57	No	2.2	0.3	0.79	No
1.0E+01	3.9	3.3	0.04	Yes	2.1	0.0	0.97	No
1.2E+01	2.1	0.1	0.96	No	2.2	0.3	0.79	No
1.4E+01	3.4	0.5	0.63	No	2.1	0.2	0.86	No
1.6E+01	3.0	1.0	0.44	No	2.2	0.3	0.80	No
1.8E+01	2.4	0.4	0.76	No	2.2	0.3	0.82	No
2.0E+01	2.0	0.1	0.94	No	2.2	0.2	0.88	No
2.2E+01	3.1	0.6	0.58	No	2.1	0.2	0.88	No
2.4E+01	4.0	0.1	0.89	No	2.2	0.1	0.92	No
2.6E+01	4.0	0.0	0.99	No	2.3	0.1	0.90	No
2.8E+01	3.2	0.5	0.65	No	2.3	0.2	0.86	No
3.0E+01	3.0	0.2	0.86	No	2.2	0.2	0.88	No
3.2E+01	2.1	0.5	0.68	No	2.4	0.1	0.94	No
3.4E+01	3.9	0.4	0.74	No	2.4	0.1	0.91	No
3.6E+01	2.1	0.3	0.82	No	2.3	0.0	0.99	No
3.8E+01	3.5	0.1	0.91	No	2.5	0.1	0.91	No
4.0E+01	2.8	0.0	0.99	No	2.6	0.1	0.94	No
1.0E+02	2.9	0.3	0.79	No	3.2	0.5	0.67	No
2.0E+02	3.3	0.8	0.49	No	3.2	0.8	0.50	No
3.0E+02	3.5	0.6	0.57	No	3.6	0.7	0.56	No
4.0E+02	3.9	0.8	0.51	No	3.9	0.7	0.51	No
6.0E+02	3.3	0.9	0.42	No	3.3	0.9	0.42	No
8.0E+02	3.8	1.2	0.33	No	3.8	1.1	0.33	No
1.0E+03	4.0	3.1	0.05	No	4.0	3.1	0.05	No
1.2E+03	3.2	4.1	0.03	**Yes**	3.2	4.1	0.03	**Yes**
1.4E+03	3.1	4.6	0.02	**Yes**	3.1	4.6	0.02	**Yes**
1.6E+03	3.0	3.4	0.04	**Yes**	3.0	3.4	0.04	**Yes**
1.8E+03	3.1	3.3	0.05	**Yes**	3.1	3.3	0.05	**Yes**
2.0E+03	2.3	3.5	0.07	No	2.3	3.5	0.07	No
2.2E+03	2.3	1.5	0.27	No	2.3	1.5	0.27	No
2.4E+03	2.3	2.5	0.13	No	2.3	2.5	0.13	No
2.6E+03	2.2	3.2	0.08	No	2.2	3.2	0.08	No
2.8E+03	2.0	3.4	0.08	No	2.0	3.4	0.08	No
3.0E+03	2.0	3.0	0.10	No	2.0	3.0	0.10	No

Table S5. sPPB-H^+ polarization curves *T*-test – Reference versus US Probe. *T*-tests were performed for each data point to 95% confidence limits, where $p < 0.05$ indicates statistically different values.

	Power Density Curve				Voltage Curve			
**Current Density**	**Degrees of Freedom**	***t*-value**	***p*-value**	**Statistically Different?**	**Degrees of Freedom**	***t*-value**	***p*-value**	**Statistically Different?**
2.0E+00	2.6	2.4	0.14	No	3.5	0.8	0.49	No
4.0E+00	3.6	1.7	0.19	No	4.0	0.8	0.46	No
6.0E+00	3.0	0.4	0.72	No	3.9	1.1	0.36	No
8.0E+00	3.9	1.2	0.31	No	3.9	1.1	0.37	No
1.0E+01	4.0	2.6	0.08	No	4.0	1.3	0.29	No
1.2E+01	3.5	2.1	0.12	No	4.0	1.0	0.38	No
1.4E+01	3.7	1.5	0.22	No	3.8	1.1	0.33	No
1.6E+01	3.9	0.6	0.61	No	3.9	1.2	0.31	No
1.8E+01	3.9	0.8	0.50	No	4.0	1.2	0.32	No
2.0E+01	3.7	0.9	0.44	No	4.0	0.9	0.46	No
2.2E+01	4.0	0.5	0.64	No	3.8	0.8	0.50	No
2.4E+01	3.3	1.2	0.31	No	3.6	0.7	0.51	No
2.6E+01	3.4	0.8	0.50	No	3.8	0.7	0.52	No
2.8E+01	3.5	0.9	0.44	No	3.8	0.7	0.55	No
3.0E+01	3.4	1.2	0.30	No	3.8	0.7	0.56	No
3.2E+01	3.8	0.3	0.80	No	3.9	0.8	0.50	No
3.4E+01	3.9	1.3	0.29	No	3.8	0.7	0.54	No
3.6E+01	3.3	0.2	0.87	No	3.9	0.7	0.51	No
3.8E+01	3.1	0.9	0.42	No	3.9	0.9	0.45	No
4.0E+01	2.6	0.9	0.46	No	3.7	0.8	0.47	No
1.0E+02	4.0	0.9	0.45	No	4.0	1.0	0.39	No
2.0E+02	3.9	1.3	0.28	No	3.9	1.3	0.28	No
3.0E+02	3.6	1.6	0.22	No	3.6	1.6	0.21	No
4.0E+02	3.6	2.0	0.14	No	3.6	1.9	0.15	No
6.0E+02	3.6	2.5	0.09	No	3.6	2.5	0.09	No
8.0E+02	2.1	4.0	0.06	No	2.1	4.0	0.06	No
1.0E+03	2.7	4.8	0.04	**Yes**	2.7	4.8	0.04	**Yes**
1.2E+03	3.2	5.9	0.01	**Yes**	3.2	5.9	0.01	**Yes**
1.4E+03	3.4	5.8	0.01	**Yes**	3.4	5.8	0.01	**Yes**
1.6E+03	3.6	3.6	0.04	**Yes**	3.6	3.6	0.04	**Yes**
1.8E+03	4.0	2.5	0.09	No	4.0	2.5	0.09	No
2.0E+03	4.0	1.7	0.19	No	4.0	1.7	0.19	No
2.2E+03	3.4	0.6	0.60	No	3.4	0.6	0.60	No
2.4E+03	4.0	0.9	0.42	No	4.0	0.9	0.42	No
2.6E+03	3.9	1.0	0.38	No	3.9	1.0	0.38	No
2.8E+03	3.9	1.2	0.33	No	3.9	1.2	0.33	No
3.0E+03	3.9	1.3	0.27	No	4.0	1.3	0.27	No

Table S6. sPPB-H^+ polarization curves *T*-test – US Bath versus US Probe. *T*-tests were performed for each data point to 95% confidence limits, where $p < 0.05$ indicates statistically different values.

	Power Density Curve				Voltage Curve			
Current Density	Degrees of Freedom	*t*-value	*p*-value	Statistically Different?	Degrees of Freedom	*t*-value	*p*-value	Statistically Different?
2.0E+00	4.0	0.4	0.70	No	2.6	1.7	0.23	No
4.0E+00	3.9	0.3	0.77	No	2.2	1.6	0.24	No
6.0E+00	2.1	0.6	0.62	No	2.2	1.9	0.20	No
8.0E+00	2.5	2.5	0.13	No	2.2	1.9	0.20	No
1.0E+01	4.0	0.7	0.52	No	2.1	1.9	0.20	No
1.2E+01	2.0	2.5	0.13	No	2.2	1.7	0.22	No
1.4E+01	3.9	1.7	0.18	No	2.1	2.0	0.18	No
1.6E+01	3.2	1.3	0.28	No	2.2	2.1	0.17	No
1.8E+01	2.3	1.3	0.33	No	2.3	2.0	0.19	No
2.0E+01	2.0	1.6	0.25	No	2.1	1.3	0.32	No
2.2E+01	3.2	1.3	0.28	No	2.1	1.1	0.39	No
2.4E+01	3.4	1.3	0.28	No	2.1	1.0	0.43	No
2.6E+01	3.4	0.8	0.51	No	2.2	1.0	0.42	No
2.8E+01	2.6	1.4	0.31	No	2.2	1.0	0.42	No
3.0E+01	3.8	1.5	0.22	No	2.1	1.0	0.44	No
3.2E+01	2.1	0.7	0.55	No	2.2	1.0	0.41	No
3.4E+01	4.0	1.8	0.17	No	2.2	1.0	0.44	No
3.6E+01	2.0	0.4	0.74	No	2.2	0.9	0.44	No
3.8E+01	2.6	0.9	0.45	No	2.4	1.0	0.43	No
4.0E+01	2.1	1.0	0.43	No	2.4	0.9	0.46	No
1.0E+02	3.0	0.8	0.48	No	3.2	0.8	0.50	No
2.0E+02	3.5	0.8	0.50	No	3.5	0.8	0.47	No
3.0E+02	4.0	1.2	0.33	No	4.0	1.1	0.34	No
4.0E+02	3.9	1.3	0.28	No	3.9	1.3	0.28	No
6.0E+02	2.7	0.7	0.56	No	2.7	0.7	0.56	No
8.0E+02	2.1	1.8	0.21	No	2.1	1.8	0.21	No
1.0E+03	2.8	1.0	0.42	No	2.8	1.0	0.42	No
1.2E+03	4.0	2.6	0.08	No	4.0	2.6	0.08	No
1.4E+03	3.9	1.9	0.15	No	3.9	2.0	0.15	No
1.6E+03	3.7	0.6	0.59	No	3.7	0.6	0.59	No
1.8E+03	3.2	0.3	0.81	No	3.2	0.3	0.81	No
2.0E+03	2.2	1.1	0.39	No	2.2	1.1	0.39	No
2.2E+03	2.6	1.2	0.35	No	2.6	1.2	0.35	No
2.4E+03	2.3	1.2	0.35	No	2.3	1.2	0.35	No
2.6E+03	2.1	1.4	0.29	No	2.1	1.4	0.29	No
2.8E+03	2.0	1.3	0.32	No	2.0	1.3	0.32	No
3.0E+03	2.0	1.4	0.31	No	2.0	1.4	0.31	No

Table S7. *T*-tests for sPPB-H^+ reference, US bath, and US probe charge transfer resistance. *T*-tests were performed to 95% confidence limits, where $p < 0.05$ indicates statistically different values.

Samples	Degrees of Freedom	*t*-value	*p*-value	Statistically Different?
**sPPB-H^+ Reference versus US Bath**	4.0	1.7	0.18	No
**sPPB-H^+ Reference versus US Probe**	3.8	1.6	0.20	No
**sPPB-H^+ US Bath versus US Probe**	4.0	0.2	0.86	No

References

[1] A.G. Wright, J. Fan, B. Britton, T. Weissbach, H.-F. Lee, E.A. Kitching, T.J. Peckham, S. Holdcroft, Hexamethyl-p-terphenyl poly(benzimidazolium): a universal hydroxide-conducting polymer for energy conversion devices, Energy Environ. Sci. 9 (2016) 2130–2142. doi:10.1039/C6EE00656F.

[2] S. de la Rochebrochard d'Auzay, J.-F. Blais, E. Naffrechoux, Comparison of characterization methods in high frequency sonochemical reactors of differing configurations, Ultrason. Sonochem. 17 (2010) 547–554.

[3] T. Soboleva, X. Zhao, K. Malek, Z. Xie, T. Navessin, S. Holdcroft, On the micro-, meso-, and macroporous structures of polymer electrolyte membrane fuel cell catalyst layers, ACS Appl. Mater. Interfaces. 2 (2010) 375–384. doi:10.1021/am900600y.

[4] E. Balogun, M. Adamski, S. Holdcroft, Non-Fluorous, Hydrocarbon PEMFCs, Generating >1 W cm-2 Power, J. Electrochem. Soc. (2020) Articles ASAP. doi:10.1149/1945-7111/ab88bd/pdf.

[5] S.J. Lee, C. De Hsu, C.H. Huang, Analyses of the fuel cell stack assembly pressure, J. Power Sources. 145 (2005) 353–361. doi:10.1016/j.jpowsour.2005.02.057.

[6] N. Ul Hassan, M. Kilic, E. Okumus, B. Tunaboylu, A.M. Soydan, Experimental determination of optimal clamping torque for AB-PEM Fuel cell, J. Electrochem. Sci. Eng. 6 (2016) 9. doi:10.5599/jese.198.

[7] E. Alizadeh, M.M. Barzegari, M. Momenifar, M. Ghadimi, S.H.M. Saadat, Investigation of contact pressure distribution over the active area of PEM fuel cell stack, Int. J. Hydrogen Energy. 41 (2016) 3062–3071. doi:10.1016/j.ijhydene.2015.12.057.

[8] E. Balogun, A.O. Barnett, S. Holdcroft, Cathode starvation as an accelerated conditioning procedure for perfluorosulfonic acid ionomer fuel cells, J. Power Sources Adv. 3 (2020) 100012. doi:10.1016/j.powera.2020.100012.

[9] M.C. Lefebvre, R.B. Martin, P.G. Pickup, Characterization of ionic conductivity profiles within proton exchange membrane fuel cell gas diffusion electrodes by impedance spectroscopy, Electrochem. Solid-State Lett. 2 (1999) 259–261. doi:10.1149/1.1390804.

[10] Z. Qi, P.G. Pickup, High performance conducting polymer supported oxygen reduction catalysts, Chem. Commun. (1998) 2299–2300. doi:10.1039/a805322g.

[11] A. Baricci, R. Mereu, M. Messaggi, M. Zago, F. Inzoli, A. Casalegno, Application of computational fluid dynamics to the analysis of geometrical features in PEM fuel cells flow fields with the aid of impedance spectroscopy, Appl. Energy. 205 (2017) 670–682. doi:10.1016/j.apenergy.2017.08.017.

[12] T. Holmes, Reaction of Hydroxyl Radicals with Sulfonated Phenylated Polyphenylenes, Simon Fraser University, 2019.
